# Integrative Multiparametric Analysis of Circulating Cell‐Free Nucleic Acids of Plasma in Healthy Individuals During Aging

**DOI:** 10.1111/acel.70133

**Published:** 2025-06-19

**Authors:** Nicolas P. Tessier, Lise M. Hardy, Florence Mauger, Antoine Daunay, Christian Daviaud, Ilef Hchaichi, Caroline Horgues, Mourad Sahbatou, Hélène Le Buanec, Jean‐François Deleuze, Alexandre How‐Kit

**Affiliations:** ^1^ Laboratory for Genomics Foundation Jean Dausset—CEPH Paris France; ^2^ Commissariat à L'energie Atomique et Aux Energies Alternatives, Centre National de Recherche en Génomique Humaine Université Paris‐Saclay Gif‐sur‐Yvette France; ^3^ Saint‐Louis Research Institute, INSERM U976—HIPI Unit University of Paris Paris France

**Keywords:** aging, circulating cell‐free DNA, circulating cell‐free nucleic acids, circulating cell‐free RNA, DNA methylation, miRNA, plasma

## Abstract

Plasma circulating cell‐free nucleic acids (ccfNAs) provide an exceptional source of information about an individual's health, yet their biology in healthy individuals during aging remains poorly understood. Here, we present the first integrative multiparametric analysis of the major types of plasma ccfNAs, including nuclear (ccfnDNA) and mitochondrial (ccfmtDNA) DNA, as well as ribosomal (ccfrRNA), messenger (ccfmRNA) and micro‐RNA (ccfmiRNA) in 139 healthy donors aged 19–66 years. We focused on quantity, integrity, and DNA methylation using an optimized experimental workflow that combines highly sensitive analytical methods with the detection of highly repetitive DNA and highly abundant RNA sequences, thereby reducing the required amount of ccfNAs per analysis. We showed a highly significant increase in ccfnDNA levels during aging (*p* < 0.001), associated with a decrease in its integrity (*p* < 0.05), while no significant changes were detected in ccfmtDNA levels and ccfDNA methylation. Moreover, a significant increase in ccfmRNA and ccfrRNA (*p* < 0.05), as well as *miR‐483‐5p* (*p* < 0.001) levels was detected during aging, but without any changes in ccfRNA integrity. Finally, we also showed that ccfDNA and ccfRNA levels were correlated (*p* < 0.001), and a similar pattern was observed for ccfmtDNA and ccfRNA levels, suggesting a possible common release, maintenance, and/or clearance mechanism. Therefore, our study provides an optimized workflow for the global analysis of ccfNAs, enhances the understanding of their biology during aging, and identifies several potential ccfNA‐based biomarkers of aging.

## Graphical abstract

We performed the first integrative multiparametric analysis of plasma circulating cell‐free nucleic acids, including nuclear and mitochondrial DNA, as well as ribosomal, messenger, and micro‐RNAs, in a cohort of 139 healthy individuals. We focused on quantity, integrity, and DNA methylation using assays targeting highly repetitive DNA and highly abundant RNA.

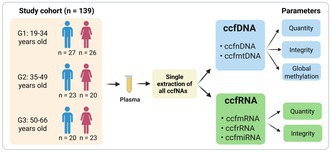

## 1 Introduction

Although circulating cell‐free nucleic acids (ccfNAs) were first described by Mandel and Metais (1948) in 1948, significant interest has only emerged in recent decades due to their potential as non‐invasive biomarkers in various diseases and health conditions, particularly ccfDNA and ccfmiRNA (Armand‐Labit and Pradines 2017; Bruno et al. 2020; Pozniak et al. 2022). Thus, since the 2000s, studies on ccfNAs from serum and plasma have increased exponentially, with plasma being the preferred matrix for analysis (Peng et al. 2024). They are now crucial tools for prenatal screening and oncology, given the high proportion of fetal and tumoral ccfNAs in the bloodstream (Szilagyi et al. 2020; Thierry et al. 2016). However, only a few studies have gone beyond the disease context to explore the biology of ccfNAs and their dynamics in healthy individuals during aging, examining inter‐individual variations that could improve their use in many applications (Tessier et al. 2023).

ccfNAs include ccfnDNA and ccfmtDNA, as well as ccfRNAs, including both coding RNA (ccfmRNA) and non‐coding RNA, such as ccfrRNA and ccfmiRNA (Tessier et al. 2023). Their forms are variable, including linear or circular, single (ss) or double‐stranded (ds). Moreover, they can be free, enclosed in extra‐cellular vesicles (EVs) (microvesicles, exosomes or apoptotic bodies), or associated with virtosomes or proteins (Thierry et al. 2016). Apoptosis is a primary mechanism for ccfNA release (Grabuschnig et al. 2020), resulting in the main mono‐nucleosome peak and the residual di‐ and tri‐nucleosome peaks, typical of ccfDNA profile (Ungerer et al. 2020). Other mechanisms can result from accidental (e.g., necrosis) or regulated deaths (pyroptosis and NETosis), and from passive (e.g., necrosis) or active (e.g., secretion) mechanisms (Aucamp et al. 2018; Grabuschnig et al. 2020; Stejskal et al. 2023; Thierry et al. 2016; Tzimagiorgis et al. 2011). The active secretion of exosomes (~50–150 nm) and microvesicles (~100–1000 nm) is implicated in long‐distance intercellular communication (Mathieu et al. 2019). These EVs contain various proteins, lipids, and nucleic acids that contribute to ccfNAs, reflecting the molecular functions and physiological conditions of the secreting cells, tissues, and/or organs in both healthy and pathological contexts (De Toro et al. 2015). They can also mediate biological functions in the targeted acceptor cells after cargo delivery, such as miRNA‐mediated mRNA silencing (Mathieu et al. 2019). Hematopoietic‐derived cells have been identified as the major contributors of plasma ccfNAs. Thus, epigenetic profiling reported leukocytes, erythrocyte progenitors, and vascular endothelial cells as the three main contributors of ccfDNA (Moss et al. 2018; Snyder et al. 2016). Platelets, erythrocyte progenitors, and leukocytes were identified as primary sources of ccfRNA from ccfRNA‐seq data (Vorperian et al. 2022), while RT‐qPCR experiments identified red blood cells for ccfmiRNA (Pritchard et al. 2012).

Due to their low concentration in the plasma of healthy individuals, most studies have been limited to analyzing a single type of ccfNA and/or a single measured parameter. Some highlighted the positive correlation of total and nuclear ccfDNA with age among adults, including nonagenarians (Jylhava et al. 2011, 2013; Li et al. 2024; Meddeb et al. 2019). Furthermore, a lower ccfnDNA level was correlated to good health, exercise, and nutritional supplementation in healthy individuals aged 65–98 years old (y.o) (Tosevska et al. 2016), while elevated ccfDNA levels were correlated with inflammatory status (Jylhava et al. 2012) and frailty (Nidadavolu et al. 2022), supporting the potential of the low ccfDNA level as a biomarker of healthy aging. Regarding ccfDNA methylation, hypomethylation of *Line1* and *Alu* retroelements has been described during aging (Erichsen et al. 2018), as well as the identification of 2000 age‐related differentially methylated CpGs (Li et al. 2024). Conversely, studies investigating ccfmtDNA levels in aging yielded conflicting results, with some showing a positive correlation (Padilla‐Sanchez et al. 2020; Pinti et al. 2014) and others reporting no association (Jylhava et al. 2013; Meddeb et al. 2019). Finally, ccfmiRNAs are the only type of ccfRNAs already studied in healthy individuals during aging. Age‐related ccfmiRNAs either increased or decreased in older individuals (Accardi et al. 2022; Ameling et al. 2015; Olivieri et al. 2014, 2012) and some showed lower levels in individuals with better health status and could potentially be biomarkers of healthy aging (Balzano et al. 2017; Morsiani et al. 2021).

Despite their promise, the study of ccfNAs in aging in healthy individuals remains limited to either ccfDNA or ccfmiRNAs. Other types of ccfRNA, including mRNA and rRNA, as well as the correlations between different ccfNA types, remain largely unexplored, although they may also carry important biological information. This constrains our understanding of the biological processes underlying ccfNAs during aging. To address this gap, we performed an integrative multiparametric analysis of both ccfDNA and ccfRNA, including miRNA, mRNA, and rRNA, in plasma samples from a cohort of 139 healthy donors aged 19–66 years divided into three age groups (G1: 19–34 years, G2: 35–49 years, G3: 50–66 years). An experimental workflow was specifically developed to enable the analysis of most ccfNA types from a single plasma sample, using several optimized high‐resolution methods and assays. This study provides a comprehensive and integrative overview of ccfNA variation in healthy individuals during aging, which might improve the understanding of the biology of ccfNAs and of their potential as biomarkers of aging.

## 2 Material and Methods

### 2.1 Ethics Statement

The study was conducted in accordance with current ethical and legal frameworks. Anonymized blood samples were obtained after informed consent from healthy donors through French blood bank, EFS (Etablissement Français du Sang, Paris, France—research agreement 15/EFS/012). All methods were performed in accordance with the recommendations of the French National Committee of Ethics.

### 2.2 Study Population

The study was conducted on 139 healthy donors from the French Blood bank (Etablissement Français du sang, Paris, France) aged between 19 and 66 y.o, including 69 women (F) and 70 men (M). EFS donors must be free of ongoing infections (tested after blood collection by the EFS for HBV, HCV, HIV, syphilis, HTLV, malaria, tetanus and Chagas disease), and have no history of cancer, chronic diseases (including insulin‐treated diabetes, inflammatory bowel disease, or autoimmune disease), or cardiovascular problems. Additional eligibility criteria can be found at: https://dondesang.efs.sante.fr/quiz. Study participants were divided into three different age groups for analysis: young (G1: 19–34 y.o., *n* = 53, 49.1% F), middle‐aged (G2: 35–48 y.o., *n* = 43, 46.5% F) and older adults (G3: 50–66 y.o, *n* = 43, 53.5% F).

### 2.3 Plasma Isolation

**FIGURE 1:**
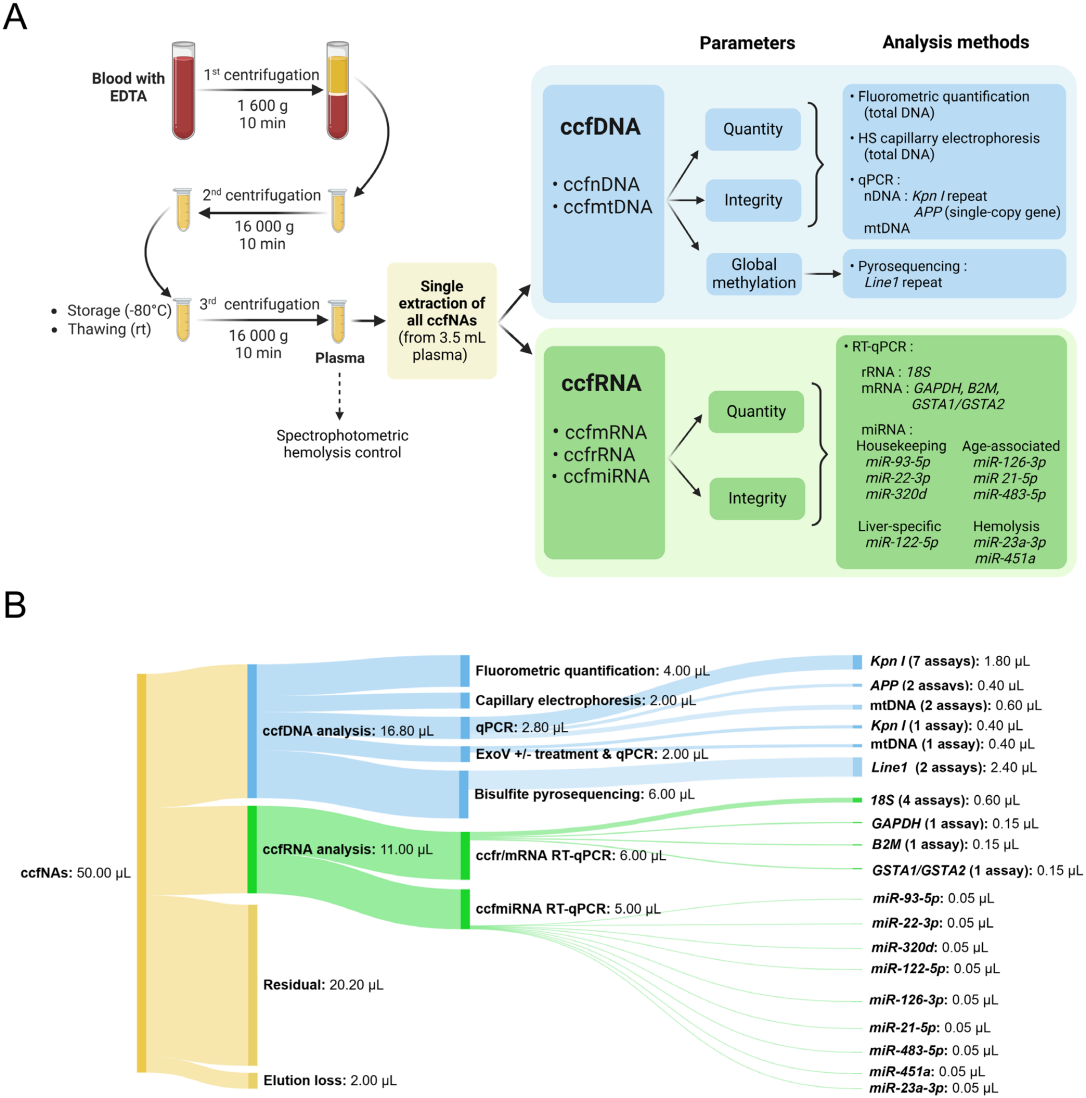
Overview of the experimental design used for the multiparametric analysis of circulating cell‐free nucleic acids from plasma. (A) Whole experimental workflow from blood collection to ccfNA analysis. (B) Sankey plot showing the volume equivalents of ccfNAs used for the different analyses in this study. ExoV, exonuclease V; HS, high‐sensitivity; rt., room temperature; RT, reverse transcription.

6 mL of blood per donor was collected between 10:00 AM and 01:00 PM in BD Vacutainer K2 EDTA tubes and was stored at room temperature from 2 to 4 h before plasma isolation. 3.8 mL of plasma was isolated by two successive centrifugations (Figure 1A). After the first centrifugation at 1,600 g for 10 min at room temperature, plasma was carefully collected to avoid collecting the buffy coat and transferred to a new tube. The second centrifugation was conducted at 16,000 *g* for 10 min at room temperature to remove any remaining leukocytes and platelets. Plasma samples were stored at −80°C in LoBind tubes (Eppendorf) prior to ccfNA extraction.

### 2.4 ccfNA Extraction

Plasma samples were thawed at room temperature and centrifuged at 16,000 *g* for 10 min at room temperature (Figure 1A). 3.5 mL of plasma was then used to isolate ccfNAs, including ccfDNAs and ccfRNAs, using the column‐based QIAamp ccfDNA/RNA kit (Qiagen) according to the manufacturer's instructions. 50 μL of H_2_O was used for elution, and the eluate was aliquoted in twin tec PCR Plates LoBind (Eppendorf) and stored at −80°C until use (2 × 24‐µL).

### 2.5 Plasma Hemolysis Measurement by UV Absorbance

Plasma hemolysis was assessed using a previously described spectrophotometric‐based method (Appierto et al. 2014), using a NanoDrop 2000c Spectrophotometer (Thermo Scientific). We first assessed the method on calibration samples made by spiking a pool of very clear non‐hemolyzed plasma samples with red blood cells (RBC) at different proportions: 0.002%, 0.004%, 0.008%, 0.0016%, 0.031%, 0.0625%, 0.125%, 0.25% and 2.5%. Spiked RBC was prepared after isolation from plasma using multiple cycles of freezing, thawing and vortex mixing. Absorbance measurement were carried out using 2 μL of plasma. Absorbances at 385 nm (A_385_) and 415 nm (A_414_) were measured to calculate the Hemolysis Score (H.S.) with the published formula: $H.S.=A_{414}-A_{385}+0.16\times A_{385}$ (Appierto et al. 2014). A H.S. threshold of 0.062 was used to consider a sample hemolyzed, calculated by the following equation: $H.S._{\text{non}-\text{hemolyzed samples}}+3\times {\mathrm{SD}}_{H.S._{\text{non}-\text{hemolyzed samples}}}$ as recommended (Appierto et al. 2014). Thus, very clear non‐hemolyzed plasma samples were used to calculate the H.S. threshold of 0.062 for hemolysis, and the calibration plasma samples spiked with 0.002%–0.0625% RBC were thereby found non‐hemolyzed.

### 2.6 Quantification of Total ccfDNA by High‐Sensitivity Fluorometry

Total ccfDNA was measured by fluorometric quantification using the Qubit dsDNA HS Assay Kit on a Qubit 3 Fluorometer (Life Technologies) according to the manufacturer's instructions. 2 μL of ccfNA was used for measurement and each sample was analyzed in duplicate. The mean value of the two measurements was used as the total ccfDNA concentration.

### 2.7 Total ccfDNA Profiling by High‐Sensitivity Capillary Electrophoresis

2 μL of total ccfDNA was analyzed by high‐sensitivity pulse‐field capillary electrophoresis using the Ultra Sensitivity NGS kit on a Femto Pulse system (Agilent), following the manufacturer's instructions. After analysis, ccfDNA profiles were obtained, and the concentrations of total ccfDNA as well as the 1st and 2nd nucleosome peaks were obtained using the calibrated ladders for quantification.

### 2.8 Primer and Probe Design and Efficiency Determination

All primers and probes are listed in Table S1, including commercial assays, previously published and newly designed primers and probes. New qPCR primers and probes were designed using Beacon Designer 8 (PREMIER Biosoft). New bisulfite sequencing PCR and pyrosequencing primers were designed using MethPrimer (http://www.urogene.org/cgi‐bin/methprimer/methprimer.cgi) and the SNP Primer design software (Qiagen), respectively. All oligonucleotides were purchased from Eurogentec using RP‐Gold purification for unmodified oligonucleotides and HPLC purification for modified oligonucleotides. All qPCR assay efficiencies were determined from standard curves generated from serial dilutions of a control sample (human leukocyte gDNA (Promega) for ccfDNA qPCR assays, control cDNA prepared from 1:1 PBMC/liver RNA (Ambion) for ccfrRNA, ccfmRNA and ccfmiRNA qPCR assays), and exhibited efficiencies between 1.72 and 2.12 (Table S1).

### 2.9 Reverse Transcription

#### 2.9.1 ccfmRNA and ccfrRNA

ccfRNA was reverse transcribed using the Quantitect Reverse Transcription Kit (Qiagen). 6 μL of input ccfNA was mixed with 2 μL of gDNA wipeout buffer and 6 μL of RNase‐free water. The mixture was incubated for 2 min at 42°C to eliminate DNA. Then, a 20‐μL reverse transcription (RT) reaction was prepared, containing the initial 14 μL mix, 1 μL of reverse transcriptase, 4 μL of RT buffer 5X, and 1 μL of RT primer mix. The RT reaction was incubated at 42°C for 30 min, followed by 3 min at 95°C to inactivate the reverse transcriptase. cDNA was stored at −20°C before ccfrRNA and ccfmRNA analysis. A control cDNA sample was also prepared from 1 μg of pooled 1:1 PBMC/liver RNA (Ambion) using the same protocol described above.

#### 2.9.2 ccfmiRNA

ccfmiRNAs were reverse transcribed using the miRCURY LNA RT Kit (Qiagen). The RT reaction contained 5 μL ccfNAPs, 2 μL 5X miRCURY SybrGreen RT reaction Buffer, 1 μL 10X miRCURY RT Enzyme, 0.5 μL UniSp6 SpikeIn (10^8^ copies/μL) and 1.5 μL RNase‐free water. The mixture was incubated for 60 min at 42°C followed by 5 min at 95°C. Samples were stored at −20°C. A control cDNA sample was also prepared from 1 μg of pooled 1:1 PBMC/liver RNA (Ambion) using the same protocol described above.

### 2.10 Quantitative PCR

Real‐time PCR was used for absolute quantification (AQ) of ccfNAs. All PCR reactions were performed in 384‐well plates (Roche) with a final reaction volume of 10 μL on a LightCycler 480 II Real‐Time PCR System (Roche). During PCR preparation, samples were always processed in 96‐well LoBind plates (Eppendorf). PCR mix and ccfNA samples were distributed in 384‐well PCR plates by a Bravo Automated Liquid Handling Platform (Agilent). ccfDNA (i), ccfmRNA, and ccfrRNA (ii), and ccfmiRNA (iii) were analyzed on separate PCR plates.

#### 2.10.1 ccfDNA, ccfmRNA and ccfrRNA Assays

ccfDNA (*Kpn I*, *APP* and mtDNA) and *18S* ccfrRNA assays were based on Syto 9 detection, while ccfmRNA assays (*GAPDH*, *B2M* and *GSTA1*/*GSTA2*) were based on hydrolysis probe detection. PCR reactions contained 1X Buffer, 1.625 mM MgCl_2_, 200 μM dNTPs, 200 nM PCR primers, 1.6 μM Syto 9 or 100 nM hydrolysis probes, 0.05 U/μL HotStarTaq DNA polymerase (Qiagen), and 2 μL of a 20‐fold dilution of ccfNA for ccfDNA analysis or 2 μL of a 4‐fold dilution of cDNA for ccfRNA analysis. Cycling conditions included an initial denaturation for 10 min at 95°C, followed by 50 cycles of amplification (95°C for 30 s, 60°C for 30 s for ccfDNA or 60 s for ccfRNA, and 72°C for 45 s), and a final melting curve analysis from 65°C to 95°C (5 acquisition per °C) to assess amplicon specificity for PCR assays based on Syto 9 detection. A control human genomic DNA (Promega) and a control cDNA sample were added to PCR plates with ccfDNA and ccfRNA assays, respectively. These samples were used as interplate calibrators for AQ.

AQ in ng/mL of plasma was performed for each ccfnDNA PCR assay using standard curves obtained from serial dilutions of human genomic DNA (Promega) ranging from 2 to 0.0002 ng/μL for *Kpn I* assays, and 9–0.0009 ng/μL for *APP* assays. Absolute concentrations were obtained using these standard curves and two interplate calibrators included in the PCR reactions, using the AQ module of the LightCycler480 Software (Roche).

AQ in copies/mL of plasma was also performed for each ccfDNA, ccfmRNA, and ccfrRNA PCR assay using standard curves generated from serial dilutions of the respective purified PCR amplicon. Briefly, a first PCR reaction was performed for each assay in a final PCR reaction volume of 20 μL, using the same concentrations and cycling conditions as above. After amplification, PCR product profiles were verified by 2% agarose gel electrophoresis and purified using AMPure XP beads (Beckman Coulter) with different bead ratios, from 1X to 3X, to eliminate unspecific PCR products. The purified PCR products were quantified in triplicate using the Qubit dsDNA HS Assay Kit (Life Technologies) and verified for their length and absence of unspecific amplification products on a Fragment Analyzer system (Agilent) using the DNF‐477 HS Small Fragment Kit, according to the manufacturer's instructions. Finally, for each amplicon, qPCR reactions were performed in duplicate using the purified PCR products serially diluted from 10 to 10, from 25,000,000 to 25 copies/μL. PCR reactions and cycling conditions were the same as above and included two PCR replicates of the interplate calibrators. The absolute concentrations of all amplicons of each ccfNA sample were calculated using these standard curves and the interplate calibrators using the AQ module of the LightCycler480 Software (Roche). Each calculated amplicon copy number was thereby reported per mL of plasma.

#### 2.10.2 ccfmiRNA Assays

10 miRCURY LNA miRNA PCR Assays (Qiagen) were used for ccfmiRNA analysis (Table S1). PCR reactions were prepared using the miRCURY LNA SYBR Green PCR Kit (Qiagen). The PCR mix contained 3 μL of 30‐fold diluted cDNA, 1X miRCURY SYBR Green Master Mix, 1 μL miRCURY LNA miRNA PCR assay, and 1 μL RNase‐free water in a final volume of 10 μL. Cycling conditions included an initial denaturation for 2 min at 95°C, followed by 50 cycles of 10 s denaturation at 95°C, a combined annealing/elongation step at 56°C for 1 min, and a final melting curve analysis from 65°C to 95°C (5 acquisition per °C) to assess amplicon specificity for SYBR detection PCR assays.

AQ of each miRNA in copies/mL of plasma was performed using the Ct value obtained with the UniSp6 spike‐in. Due to the known and fixed quantity of this synthetic small RNA spiked in each ccfNA sample before reverse transcription and to miRNA assay efficiencies that were close to 100% (Table S1), it was possible to calculate the quantity of each miRNA in a sample using the following formula: $\text{miRNA quantity}=\text{quantity of UniSp}6\times 2^{{\mathrm{Ct}}_{\text{miRNA}}-{\mathrm{Ct}}_{\text{UniSp}6}}$. Relative expression of age‐associated miRNAs (*miR‐21‐5p*, *miR‐126‐3p* and *miR‐483‐5p*), and liver‐specific *miR‐122‐5p* could also be calculated by normalizing their concentrations by the geometric mean of the concentrations of the three housekeeping‐miRNA, that is, *mir‐93‐5p*, *miR‐22‐3p*, and *miR‐320d*.

Plasma hemolysis was also evaluated by qPCR using *miR‐451a* and *miR‐23a‐3p* Ct values. *miR‐23a* is not affected by hemolysis, while *miR‐451a* is enriched in erythrocytes (Smith et al. 2022). For each sample, we calculated the Ct_*hsa‐miR‐23a‐3p*_−Ct_*hsa‐miR‐451a*_ value. A difference higher than 7 indicated hemolysis (Smith et al. 2022). This miR‐based delta Ct method recommended by Qiagen was compared to the hemolysis score (H.S.) based on plasma UV absorbance (Appierto et al. 2014) (Figure S1A,D). Using calibration plasma samples spiked with increasing percentages of red blood cells, both methods detected hemolysis and were strongly correlated (Figure S1B). However, the qPCR method proved more sensitive when using the recommended threshold. Applied to our samples, the H.S. correlated positively with Ct_*miR‐23a‐3p*_−Ct_*miR‐451a*_ and the ratio of *miR‐451a*/*miR‐23a‐3p* copies/mL, but not with each miRNA Ct or concentration alone (Figure S1C,D). Using the miRNA‐based method, six samples showed Ct_*miR‐23a‐3p*_−Ct_*miR‐451a*_ > 7 and were excluded for downstream analyses (Figure S1C).

### 2.11 Integrity Analysis

The integrity of ccfDNA and ccfRNA was determined by calculating the integrity indexes from qPCR results, and also from capillary electrophoresis data for ccfDNA. The qPCR integrity index was defined as the ratio of a large amplicon quantity to the smallest. Integrity values close to 1 indicated unfragmented ccfNAs, whereas values close to zero indicated fragmented ccfNAs. For capillary electrophoresis, the 1st and the 1st + 2nd peak concentrations were divided by the total amount of ccfDNA to calculate the integrity indexes. Integrity indexes close to 0 indicated unfragmented ccfNAs, while values close to 1 indicated fragmented ccfNAs.

### 2.12 DNA Methylation Analysis by Pyrosequencing

Bisulfite conversion of ccfDNA was performed using 6 μL of plasma ccfNAs and the EZ‐96 DNA Methylation‐Gold Kit (Zymo Research), according to the manufacturer's instructions. Converted ccfDNA was stored in LoBind plates at −20°C before use. For optimization experiments, DNA methylation standards with 0%, 25%, 50%, 75%, and 100% DNA methylation levels and concentrations of 0.01, 0.1, 1, and 10 ng/μL were prepared using converted methylated and unmethylated human DNA from the EpiTect PCR Control DNA Set (Qiagen). Two *Line1* amplicons were amplified in a 25 μL reaction by standard PCR on a LightCycler 480 Real‐Time PCR System (Roche).

PCR reactions contained 2 μL converted ccfDNA (or 1 μL of DNA methylation standard), 1X Buffer, 1.625 mM MgCl_2_, 200 μM dNTPs, 200 nM PCR primers, 1.6 μM Syto 9, and 0.08 U/μL Hot Start Taq DNA polymerase (Qiagen). Cycling conditions included an initial 10‐min denaturation at 95°C, followed by 45 cycles of amplification (30 s denaturation at 95°C, 30 s primer annealing at 60°C, and 30 s elongation at 72°C for), and a final elongation step at 72°C for 5 min. PCR products were stored at −20°C before sequencing. 10 μL of PCR products were purified using a previously described protocol (How‐Kit et al. 2015). Pyrosequencing was performed using the PyroMark Gold SQA Q96 Kit (Qiagen) on a PyroMark Q96 MD (Qiagen) using pyrosequencing sequences (Table S1), and the DNA methylation data were analyzed with PyroMark CpG software (Qiagen). We excluded one, seven, and thirteen DNA methylation outliers for CpGs 1, 2, and 4, respectively, as their DNA methylation percentages were close to or equal to 100%, which is much higher than the 100% DNA methylation standard.

### 2.13 Statistical Analysis

All analyses were performed using GraphPad Prism version 10.2.3 for Windows (GraphPad Software) and R (GPL) for PCA analysis. Correlation analysis was performed using the Pearson test with the associated *r* correlation coefficient and *p*‐values. Significant differences between groups were assessed using the Mann–Whitney *U* test, either by comparing age groups of women, men, and all individuals pairwise, or by considering both sexes within the same age range. *p*‐values are two‐sided, and *p*‐value < 0.05 is considered significant; * < 0.05, ** < 0.01, *** < 0.001, **** < 0.0001. PCA analysis was performed using the *FactoMineR_2.3* package using reduced centered data from our analysis. Missing values were replaced by the median of the variable in the data matrix. ccfmiRNA quantifications were included for hemolyzed plasma samples to avoid missing values in the data matrix.

## 3 Results

### 3.1 Description of the Overall Strategy Used for Multiparametric Analysis

We optimized an experimental workflow for the comprehensive analysis of variations in several plasma ccfNA parameters occurring during aging. It included two centrifugation steps for plasma isolation from EDTA blood before –80°C storage, and one centrifugation after thawing before the simultaneous isolation of all ccfNAs using 3.5 mL of plasma and the QIAamp ccfDNA/RNA kit (Figure 1A). To drastically reduce the amount of ccfNAs required for each analysis without losing analytical sensitivity, especially for the longest amplicons, we targeted highly repetitive DNA (*Kpn I*, *Line1* and mtDNA) and highly abundant RNA (*18S* rRNA and mRNA from housekeeping genes) sequences. Highly sensitive (HS) fluorometric quantification was used to assess total ccfDNA quantity, while HS capillary electrophoresis was used to assess both its quantity and integrity. Global ccfDNA methylation was measured by pyrosequencing, and qPCR assays were used to quantify and assess the integrity of both ccfnDNA and ccfmtDNA. RT‐qPCR was used to quantify ccfrRNA, ccfmRNA, and ccfmiRNA, and assess the integrity of ccfRNA. Each sample was also controlled for hemolysis, either directly on the plasma or by RT‐qPCR using two specific miRNAs. The different volumes of ccfNAs used for each analysis are given in Figure 1B.

### 3.2 Comparison of ccfDNA Quantification Methods

The quantification of ccfDNA is a critical step in the analysis workflow and could sometimes be challenging (Mauger et al. 2015; Ungerer et al. 2020). We therefore measured and compared ccfDNA concentrations using three reference methods: dsDNA fluorometric quantification (Qubit fluorometer), high‐sensitivity pulse‐field capillary electrophoresis (Femto Pulse), and qPCR assays targeting a small segment (60–67 bp) of either *APP*, a single‐copy gene, or *Kpn I* repeats (Figure S2A). ccfDNA quantifications exhibited mean values of 4.18 ± 2.04 , 4.79 ± 3.23 , and 2.30 ± 1.45/2.25 ± 1.47 ng per mL of plasma with Qubit, Femto Pulse, and *Kpn I*/*APP* qPCR quantification, respectively. All correlations were highly significant (2.43×10−41≤p≤2.32×10−4), with strong correlations (*r* > 0.5) found between the three quantification methods, except for the Femto Pulse and *APP* qPCR assay, exhibiting moderate correlation (*r* = 0.3) (Figure S2B). Interestingly, the best correlation (*r* = 0.88, *p* = 2.43 × 10^−41^) was found between the Qubit and *Kpn I* qPCR quantification, and not between the two qPCR assays, suggesting that *Kpn I* qPCR and Qubit could be used interchangeably for ccfDNA quantification. Notably, an overestimation of ccfDNA concentration was generally found with the Femto Pulse compared to the other methods, and also with the Qubit compared to PCR assays. qPCR assays quantified amplifiable ccfDNA of a minimum length and should always present lower values compared to whole DNA quantification methods. This was also clearly illustrated by the quantifications performed with the other qPCR assays amplifying larger DNA fragments (Figure S3).

### 3.3 Levels of ccfnDNA, but Not ccfmtDNA Levels, Increase With Age and Decrease in Integrity

**FIGURE 2:**
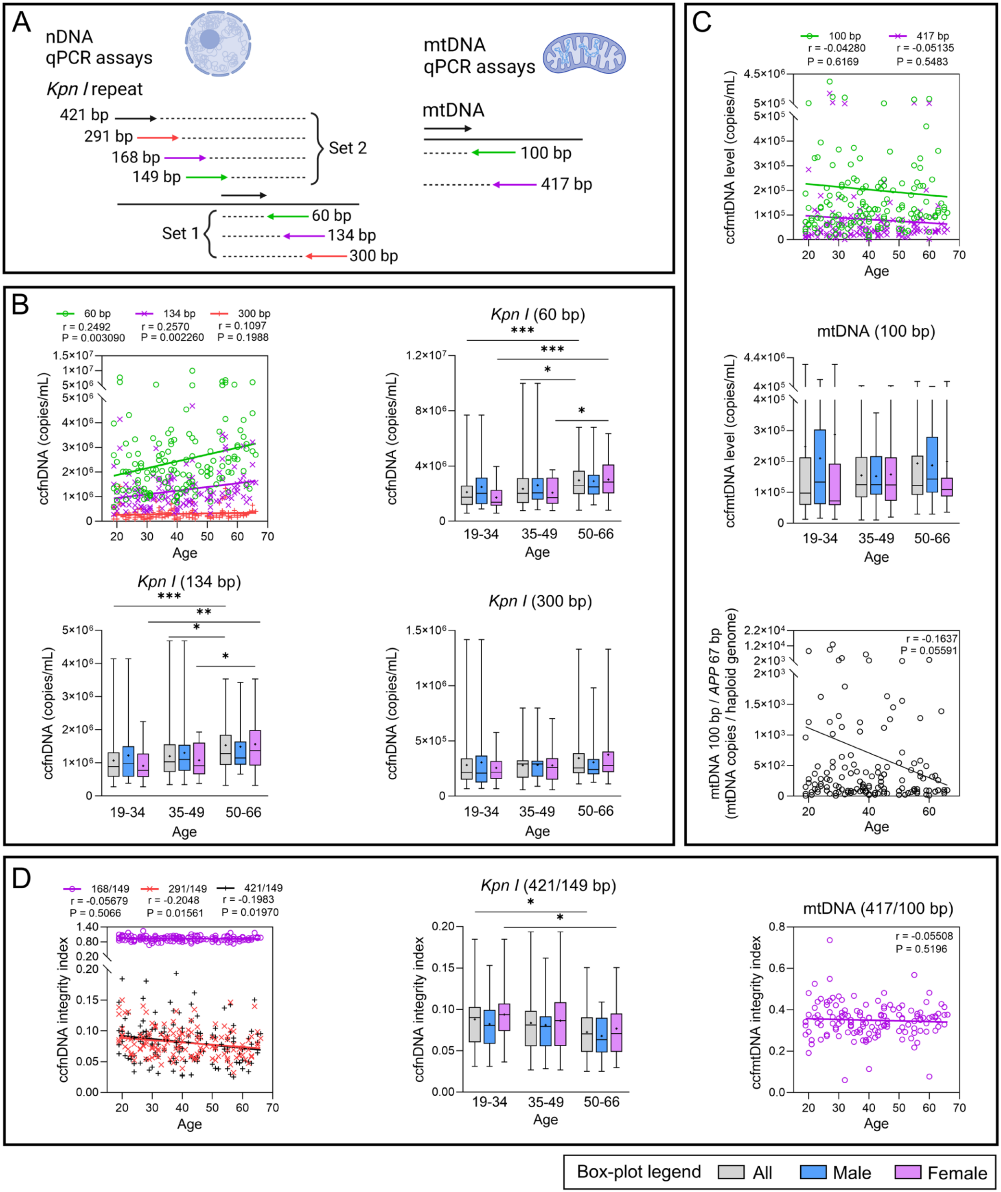
Plasma ccfDNA quantity and integrity variation during aging (*n* = 139). (A) ccfnDNA and ccfmtDNA qPCR assays used. (B) ccfnDNA quantification using Set 1 *Kpn I* repeat assays. (C) ccfmtDNA quantification. (D) ccfDNA integrity assessment using the integrity index based on the ratio of a large PCR amplicon to the smallest one. Mann–Whitney *U* tests were performed in box‐plots between each age group and between men and women of a same age group. The ‘+’ symbol indicates the mean value obtained for each group. Pearson's *r* coefficients and associated *p*‐values as well as linear regression lines are shown in scatterplots. *p*‐values < 0.05 are considered significant; * < 0.05, ** < 0.01, *** < 0.001.

Absolute quantification was performed using different sets of qPCR assays amplifying DNA sequences of different lengths ranging from 60 to 421 bp and 100–417 bp for ccfnDNA and ccfmtDNA, respectively (Figure 2A). Copy number analysis showed a slight increase in nuclear ccfDNA level between 19 and 66 y.o. with each assay, which was only significant for the smallest *Kpn I* amplicons (60–168 bp, 0.002 < *p* < 0.02, Figures 2B and S4A,B). Using the 60‐bp *Kpn I* assay, values ranged from 5.83 × 10^5^ to 9.99 × 10^6^, with a mean value of 2.45 × 10^6^ copies per plasma mL (RSD = 61.25%). When comparing ccfnDNA levels considering the three age groups, the most significant change (*p* = 0.0003) was found with the 60‐bp assay between G3 and the younger group. This significant difference was also found in women (*p* = 0.0003) but not in men when the sex was considered. Similar differences were also found with the larger assays, but their significance gradually disappeared with increasing length of the amplicons (Figures 2B and S4B).

We also analyzed quantification data obtained with other methods, which yielded similar results to those obtained with the *Kpn I* repeat. Thus, all methods showed an increase in ccfDNA levels with age, although only Qubit quantification reached significance (*p* = 0.012) in the correlation analysis (Figures S4C and S5). Furthermore, significant differences were found between G1 and G3 (all, *p* = 0.0026, and women, *p* = 0.0076) using the *APP* 67‐bp qPCR assay and Qubit quantification (Figures S4C and S5). In contrast, Femto Pulse quantification, using either the first peak, the first two peaks, or the total ccfDNA amount, showed a significant increase only in women.

Contrary to ccfnDNA, the two qPCR assays targeting ccfmtDNA showed no significant variation in their copy number with age in correlation analyses or group comparisons (Figure 2C). Calculation of the ccfmtDNA copy number per genome equivalent using the 100‐bp mtDNA and the 67‐bp *APP* assays revealed highly variable values ranging from 8 to 12,903 copies, with a mean value of 696 copies per genome equivalent (RSD = 240.5%). Moreover, a clear and nearly significant decrease in ccfmtDNA copies per genome equivalent (*r* = −0.16, *p* = 0.056) was observed during aging (Figure 2C). The levels of linear and circular ccfmtDNA were also assessed following Exonuclease V treatment of ccfNAs. Despite a median ratio between the two forms close to 1, there was high inter‐individual variability, with a few individuals presenting only one form of ccfmtDNA (Figure S6A,B). The proportion of circular ccfmtDNA showed significant positive correlations with total ccfmtDNA levels (*r* = 0.23, *p* = 0.011) and copy number per genome equivalent (*r* = 0.26, *p* = 0.0039, Figure S6C). Circular mtDNA also exhibited a decreasing trend with age in both mtDNA levels and copies per genome equivalent (Figure S6D,E). Of note, buffy coat mtDNA copies per genome equivalent did not vary with age or correlate with plasma values (Figure S7).

We also assessed ccfDNA integrity using an integrity index based on the ratio of a large amplicon to the smallest from each set of qPCR assays (Figure 2A). *Kpn I* 421/149 and 249/149 integrity indexes showed a significant decrease with age (*r* = −0.20, *p* = 0.0197 and *r* = −0.21, *p* = 0.0156, respectively), which was not visible with the 168/149, 134/60, and 300/60 indexes (Figures 2D and S4B). The decrease in the 421/149 integrity index was significant in G3 compared to G1, whether all individuals (*p* = 0.0148) or only women (*p* = 0.0455) were considered (Figures 2D and S4B), while the 134/60 and 300/60 integrity indexes were significantly higher in young women than in young men (*p* = 0.0224 and *p* = 0.0175, respectively). On the contrary, this loss of integrity was not observed for ccfmtDNA during aging (Figure 2D). We also evaluated the ability of the Femto Pulse to assess ccfDNA integrity by calculating an integrity index using the ratio of the 1st and/or 2nd peak to the total ccfDNA concentration (Figure S8). The 1st peak/Total and 1st + 2nd peaks/Total integrity indexes significantly correlated with age (*r* = 0.28, *p* = 0.00096 and *r* = 0.25, *p* = 0.003, respectively), indicating a loss of ccfDNA integrity during aging. Age‐group comparisons showed significant differences in integrity indexes between G3 and G1 (all: *p* = 6.9 × 10^−5^ and *p* = 0.0003, respectively; and women: *p* = 0.0013 and *p* = 0.0012, respectively) and sometimes revealed significant sex differences (Figure S8).

### 3.4 Global ccfDNA Methylation Did Not Vary During Aging

**FIGURE 3:**
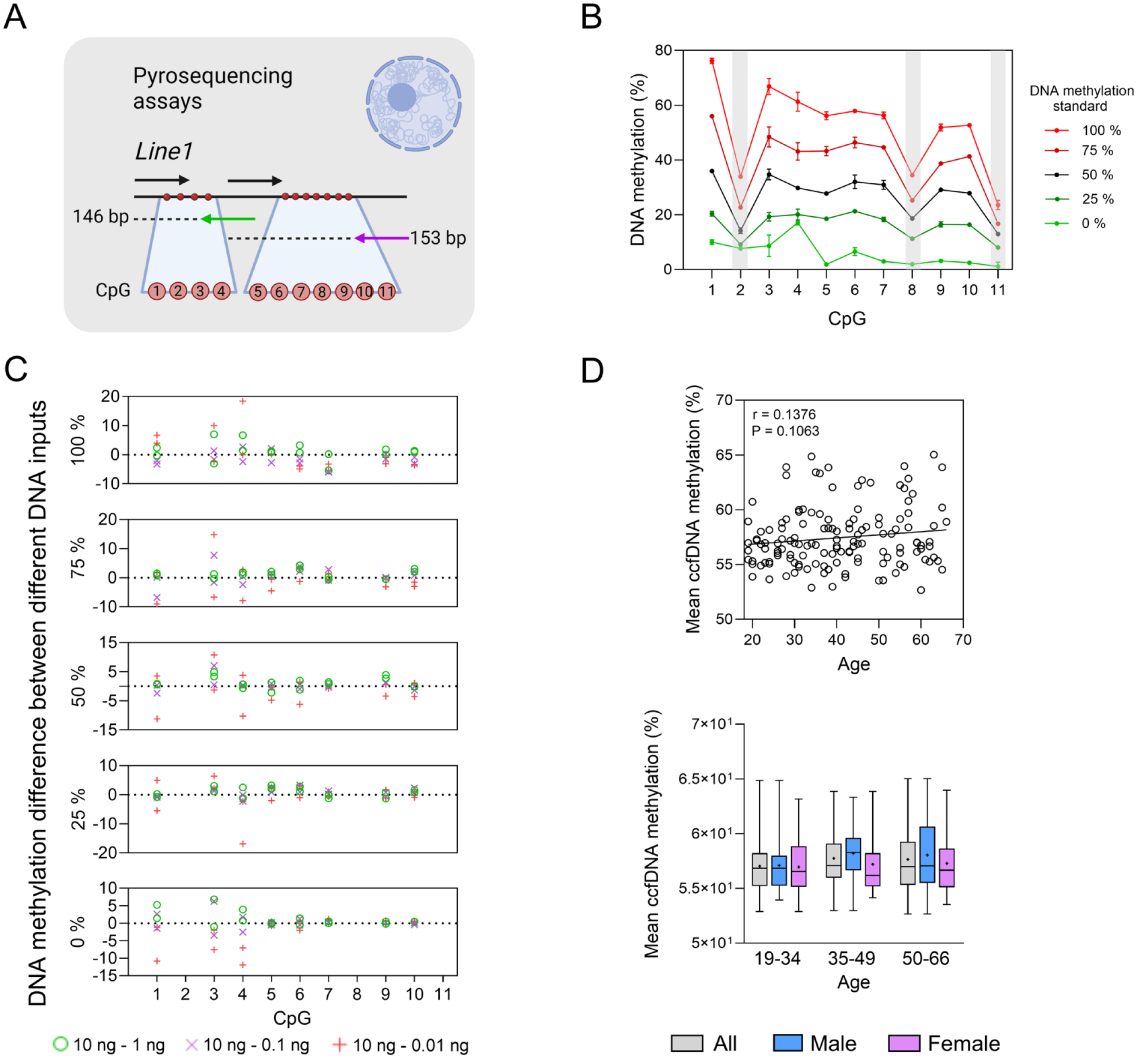
Global plasma ccfDNA methylation variation during aging. (A) *Line1* pyrosequencing assays. (B) DNA methylation profile obtained by pyrosequencing using 10 ng of DNA methylation standards ranging from 0 to 100% methylation (duplicate experiments). Shaded gray bands indicate excluded CpGs. (C) *Line1* DNA methylation difference calculated between two different quantities of DNA methylation standards used as input (duplicate experiments). (D) Global ccfDNA methylation variation during aging measured using the mean DNA methylation value of *Line1* (CpG_1,3–7,9–10_) (*n* = 139). Mann–Whitney *U* tests were performed in box‐plots between each age group and between men and women of the same age group. The ‘+’ symbol indicates the mean value obtained for each group. Pearson's *r* coefficients and associated *p*‐values, as well as linear regression lines, are indicated in scatterplots. *p*‐values < 0.05 are considered significant; * < 0.05, ** < 0.01, *** < 0.001.

To assess the compatibility of our protocol with minute and degraded DNA, we optimized two *Line1* repeat PCR and pyrosequencing assays for global ccfDNA methylation estimation using decreasing amounts (10, 1, 0.1, and 0.01 ng) of DNA methylation standards ranging from 0% to 100% (Figure 3A,B). *Line1* DNA methylation profiles obtained with 10 ng of template DNA revealed three CpGs (CpG_2_, CpG_8_, and CpG_11_) with reduced DNA methylation percentages, which were excluded from all analyses. Analysis of the eight remaining CpGs showed that picogram quantities of DNA had little effect on the DNA methylation levels (Figure 3C), indicating that the two assays should be compatible with plasma ccfDNA. Global ccfDNA methylation was thereby assessed in the entire cohort and, despite a weak positive correlation (*r* = 0.1376), showed no significant changes during aging from correlation analyses and in age and sex group comparisons (Figure 3D). Considering each CpG separately, a significant increase in ccfDNA methylation was observed in G3 compared to G1 with CpG_3_ (*p* = 0.0174) and in G2 men compared to G2 women with CpG_1_ (*p* = 0.0119, Figure S9). Moreover, when analyzing buffy coat samples, *Line1* DNA methylation did not show any correlation with age or plasma values (Figure S10). Overall, no change in global ccfDNA methylation was observed during aging.

### 3.5 Levels of ccfRNA Increase With Age but Do Not Vary in Integrity

**FIGURE 4:**
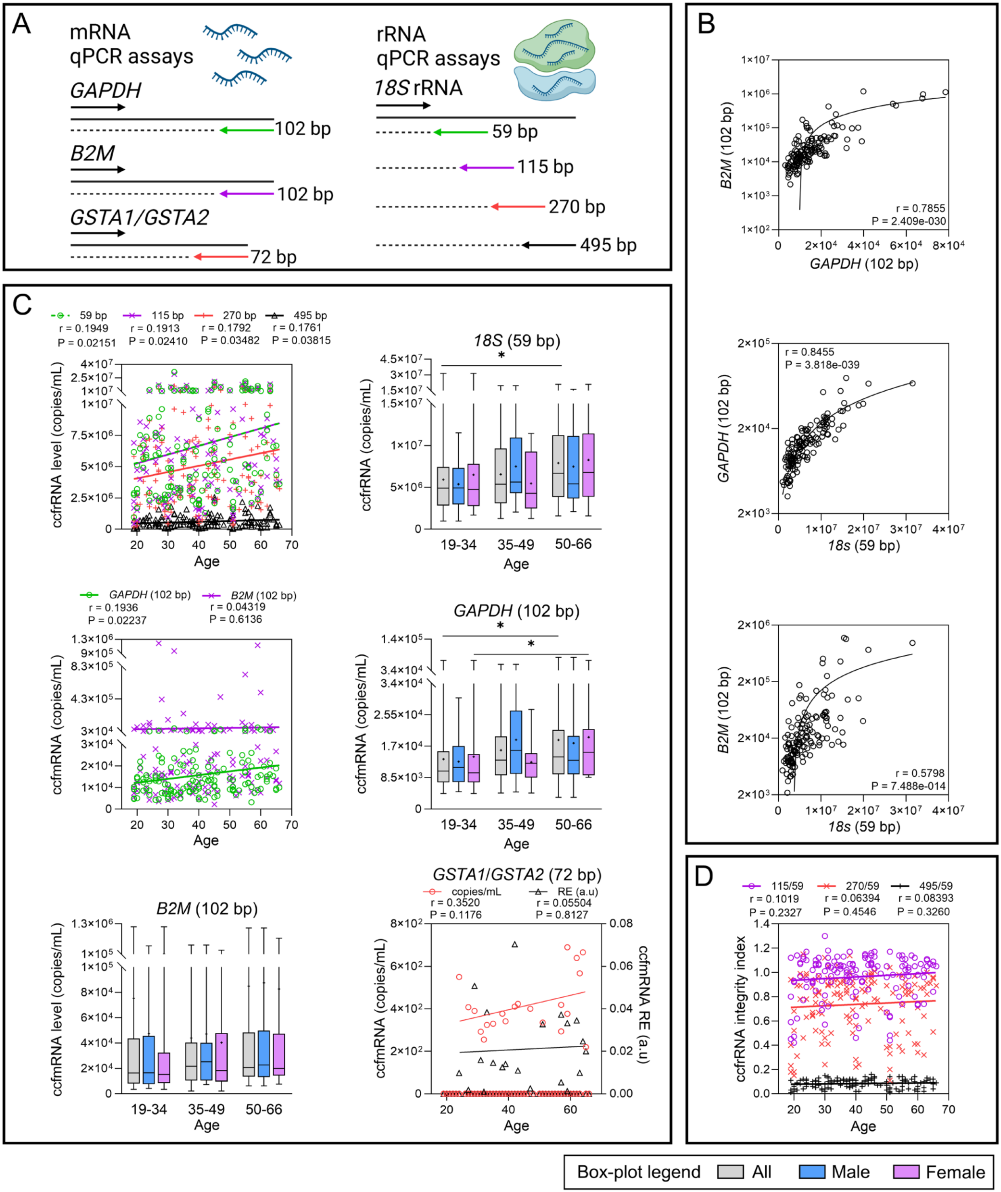
Variation of plasma ccfRNA quantity and integrity during aging (*n* = 139). (A) mRNA and rRNA qPCR assays used for ccfRNA quantification. (B) Correlation of *B2M* ccfmRNA, *GAPDH* ccfmRNA, and *18S* ccfrRNA quantity in plasma. (C) Variation of *18S* ccfrRNA and *B2M*, *GAPDH*, and *GSTA1*/*GSTA2* ccfmRNAs during aging. (D) Assessment of ccfrRNA integrity using the integrity index based on the ratio of a large *18S* amplicon to the smallest one. Mann–Whitney *U* tests were performed in box‐plots between each age group and between men and women of the same age group. The ‘+’ symbol indicates the mean value obtained for each group. Pearson's *r* coefficients and associated *p*‐values, as well as linear regression lines, are indicated in scatterplots. *p*‐values < 0.05 are considered significant; * < 0.05, ** < 0.01, *** < 0.001.

Analysis of plasma ccfRNA abundance and integrity was performed using RT‐qPCR assays targeting *18S* rRNA and four mRNAs (Figure 4A). We used *GAPDH* and *B2M*, two ubiquitously expressed housekeeping genes, as proxies for total ccfmRNA load, and also evaluated the mRNA levels of *GSTA1*/*GSTA2*, which are specifically expressed in the liver, kidney, and adrenal glands (Mlakar et al. 2021; Ng et al. 2021).

The results first showed significant moderate to strong linear correlations (0.57 < *r* < 0.84, 3.8 × 10^−39^ < *p* < 7.5 × 10^−14^) between *GAPDH* mRNA, *B2M* mRNA, and *18S* rRNA concentrations (Figure 4B), indicating that plasma ccmRNA and ccfrRNA levels are closely related. The number of *18S* rRNA, *GAPDH*, and *B2M* mRNA copies per plasma mL were also very variable among individuals, from 9.50 × 10^5^ to 3.15 × 10^7^ (59‐bp assay), 3.18 × 10^3^–7.86 × 10^4^, and 2.17 × 10^3^–1.19 × 10^6^, with a mean value of 6.73 × 10^6^ (RSD = 70.82%), 1.59 × 10^4^ (RSD = 77.02%) and 6.87 × 10^4^ (RSD = 262.5%), respectively. *18S* rRNA quantification results with the four assays revealed a significant increase with age (0.176 < *r* < 0.195 and 0.021 < *p* < 0.039), while age‐group comparisons were significant between G1 and G3 using the 59 (*p* = 0.0195) and 115‐bp (*p* = 0.0339) assays (Figures 4C and S11A). A significant increase was also observed for *GAPDH* ccfmRNA (*r* = 0.19, *p* = 0.02) but not with *B2M* ccfmRNA during aging (Figure 4C). This increase in *GAPDH* ccfmRNA level was significant between the youngest and oldest groups, whether all individuals (*p* = 0.012) or only women (*p* = 0.021) were considered. Furthermore, by targeting *GSTA1* and *GSTA2* in a single assay, we were able to detect these transcripts in 16.5% of the plasma samples. Depending on their expression level, either expressed in relative expression or in copies/mL (Figure 4C), no change or a slight increase (*r* = 0.35) was observed during aging, respectively. Despite their low abundance, ccfmRNAs from minor contributing organs can be detected in plasma, suggesting potential applications as surrogate, non‐invasive biomarkers of internal tissues and organs, particularly in the context of disease. Integrity analysis was also performed with *18S* rRNA assays using the integrity index (Figures 4D and S11B). In contrast to ccfDNA, no significant correlation was found between ccfrRNA integrity and aging, regardless of the combination of assays used.

### 3.6 Evaluation of Age‐Related and Liver‐Specific ccfmiRNA Levels in Plasma

We assessed the expression of several housekeeping (*mir‐93‐5p*, *miR‐22‐3p*, and *miR‐320d*) (Faraldi et al. 2019; Korma et al. 2020), age‐associated (*miR‐126‐3p*, *miR‐21‐5p*, *miR‐483‐5p*) (Rusanova et al. 2018; Tessier et al. 2023), liver‐specific (*miR‐122‐5p*) (Hu et al. 2022) and hemolysis control (*miR‐23a‐3p* and *miR‐451a*) ccfmiRNA in plasma samples of the entire cohort. Six samples considered to be hemolyzed were excluded from our analyses (Figure S1).

**FIGURE 5:**
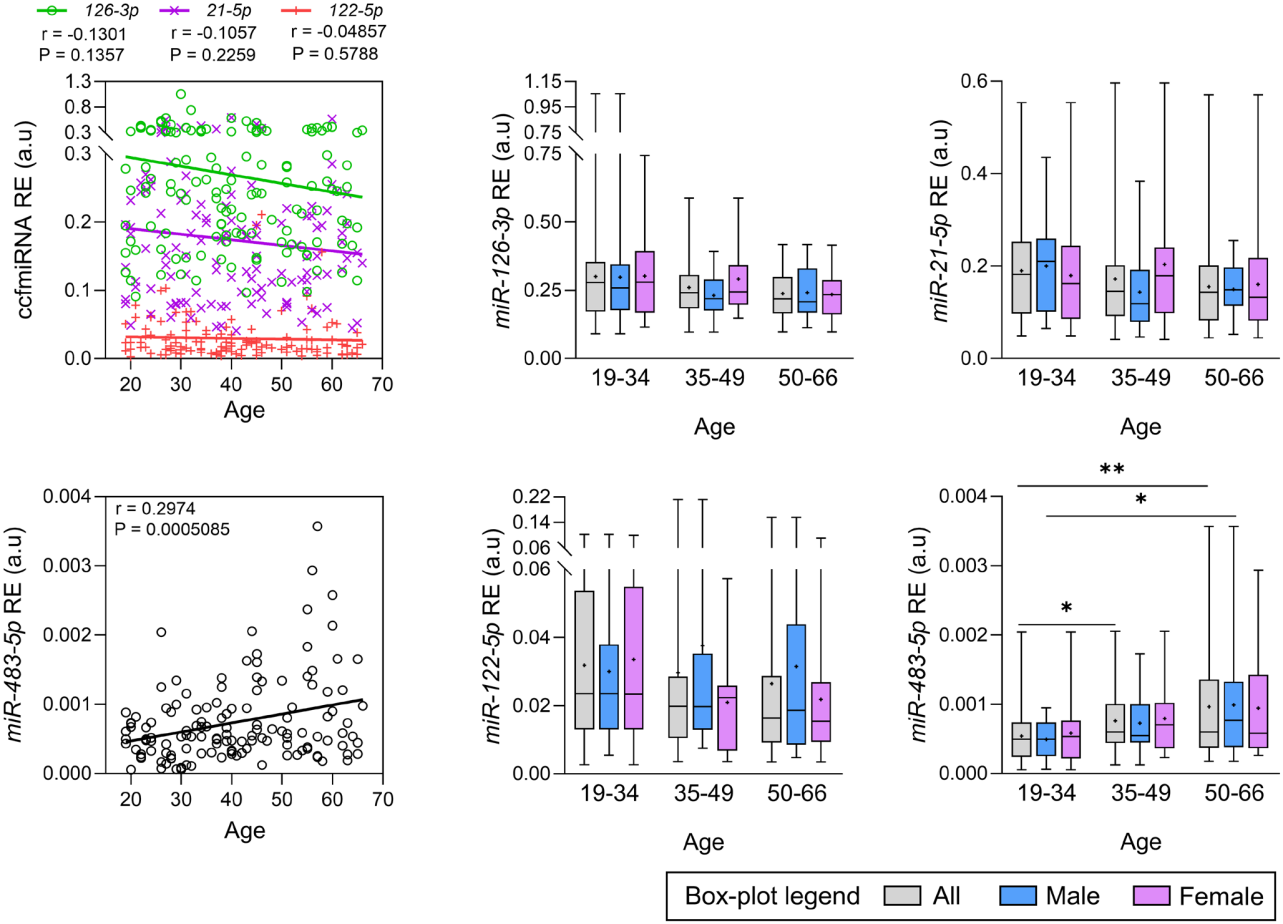
Variation in the levels of different plasma ccfmiRNAs during aging (*n* = 133 samples). Age‐associated (*miR‐126‐3p*, *miR‐21‐5p*, *miR‐483‐5p*) and liver‐specific (*miR‐122‐5p*) ccfmiRNA relative expression levels during aging. Mann–Whitney *U* tests were performed in box‐plots between each age group and between men and women of the same age group. The ‘+’ symbol indicates the mean value obtained for each group. Pearson's *r* coefficients and associated *p*‐values, as well as linear regression lines, are shown in scatterplots. *p*‐values < 0.05 are considered significant; * < 0.05, ** < 0.01, *** < 0.001.

Absolute quantification analysis revealed that the levels of housekeeping miRNAs (*mir‐93‐5p*, *miR‐22‐3p*, and *miR‐320d*) did not vary with age, as expected (Chen et al. 2020) (Figure S12A). These miRNAs were subsequently used as normalizers to calculate the relative expression of other miRNAs. Among the age‐related ccfmiRNAs tested, only *miR‐483‐5p* correlated significantly with age using either absolute quantification (*r* = 0.196, *p* = 0.024, Figure S12B) or relative expression (*r* = 0.2974, *p* = 5.1 × 10^−4^, Figure 5). Group comparisons showed significantly higher concentrations in G3 compared with G1 for all individuals (*p* = 0.0089) and for men (*p* = 0.032). Liver‐specific *miR‐122‐5p* was detected in all plasma samples and showed significantly lower levels in G2 compared to G1, for both all adults (*p* = 0.0374) and women (*p* = 0.0284), but only when considering absolute concentration (Figure S12C).

As our targeted RT‐qPCR approach may have missed several age‐associated miRNAs, we performed miRNA‐sequencing of ccfmiRNAs from 24 additional plasma samples: 12 from young adults (29–39 years old) and 12 from older adults (50–68 years old). Differential expression analysis identified 32 over‐expressed and 15 under‐expressed miRNAs in older compared to younger individuals, using thresholds of 2‐fold expression change and *p* < 0.05 (Figure S13 and Table S2). Of these, 4 (*miR‐30b‐5p*, *miR‐885‐5p*, *miR‐23b‐3p* and *miR‐3659*) and 1 (*miR‐20a‐3p*), respectively, remained significant after applying a 10% FDR correction. Notably, among the three candidate age‐associated miRNAs investigated, only *miR‐483‐5p* was significantly increased in older individuals (*p* = 0.00993, without FDR correction), which confirms our RT‐qPCR results.

### 3.7 ccfDNA and ccfRNA Levels Are Correlated but Display Different Patterns

**FIGURE 6:**
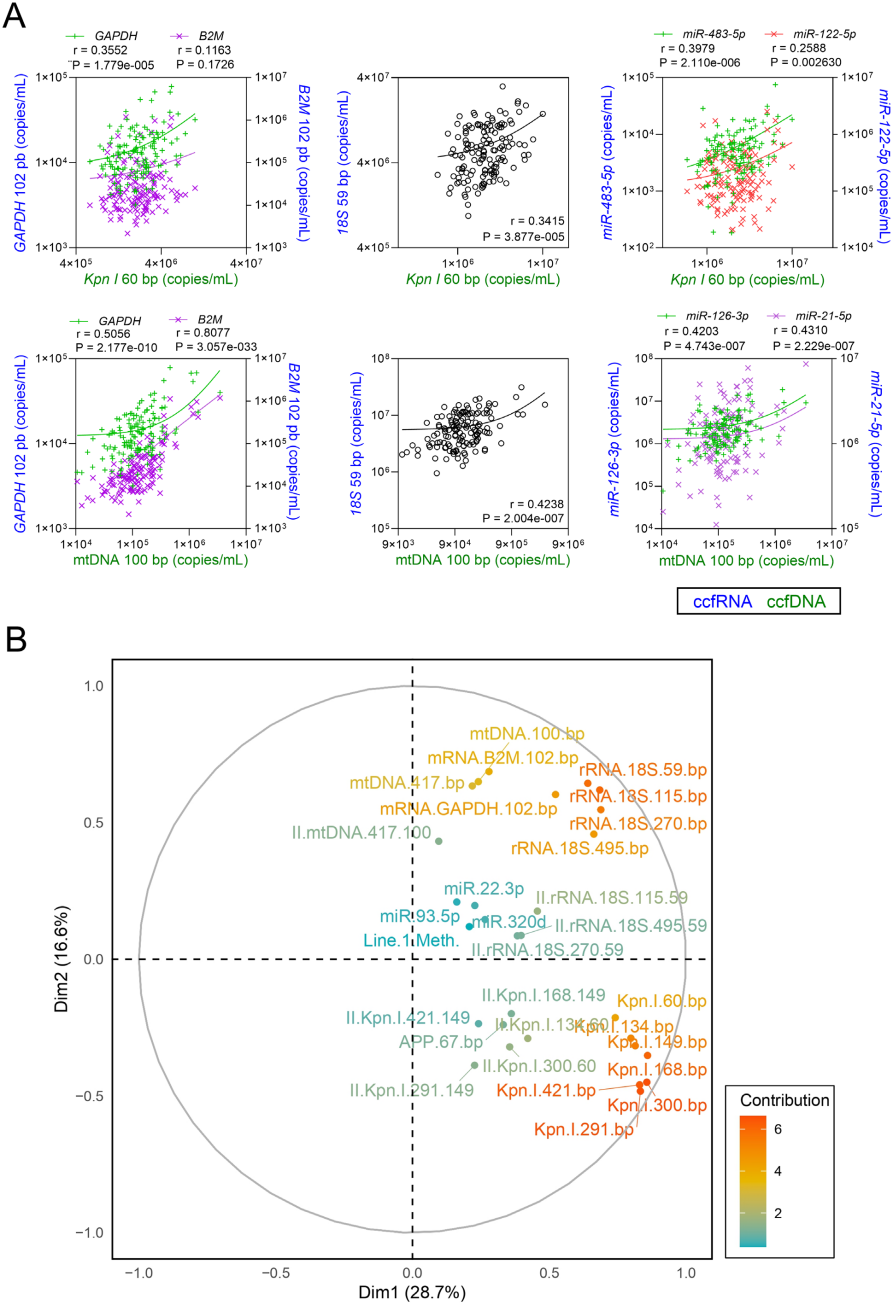
Comparison of ccfDNA and ccfRNA variations in plasma (*n* = 139 samples). (A) Correlation of ccfnDNA and ccfmtDNA, using *Kpn I* 60 bp and mtDNA 100 bp assays respectively, with ccfmRNA (*GAPDH* 102 bp, *B2M* 102 bp), ccfrRNA (*18S* 59 pb), and ccfmiRNA (*miR‐126‐3p*, *miR‐21‐5p*, *miR‐483‐5p*, *miR‐122‐5p*). Linear regression lines are shown, Pearson's *r* correlation coefficients and associated *p*‐values are indicated in each graph. *p*‐values < 0.05 are considered significant. (B) Principal component analysis of the different variables used. The contribution of the variables to the first two components is indicated by a color scale. II, integrity index.

We first investigated whether there was a correlation between the amounts of ccfDNA and ccfRNA among healthy individuals. Figure 6A shows that ccfnDNA concentrations using the *Kpn I* repeat 60‐bp assay correlated weakly but significantly with ccfmRNA *GAPDH* assay (*r* = 0.35, *p* = 1.8 × 10^−5^) and with ccfrRNA using the *18S* 59‐bp assay (*r* = 0.34, *p* = 3.9 × 10^−5^). Moreover, ccfnDNA was also significantly correlated to the only ccfmiRNA that increased significantly during aging in our study, *miR‐483‐5p* (*r* = 0.40, *p* = 2.1 × 10^−6^), and also to liver‐specific *miR‐122‐5p* (*r* = 0.26, *p* = 0.003). Using the mtDNA 100‐bp assay, ccfmtDNA concentrations correlated significantly and strongly with *B2M* ccfmRNA levels (*r* > 0.8, 3.1 × 10^−33^), and significantly but moderately with *GAPDH* ccfmRNA (*r* = 0.5, *p* = 2.2 × 10^−10^) and *18S* ccfrRNA using the 59‐bp assay (*r* = 0.42, *p* = 2 × 10^−7^). We also observed a significant correlation of ccfmtDNA with *miR‐126‐3p* (*r* = 0.42, *p* = 4.7 × 10^−7^) and *miR‐21‐5p* (*r* = 0.43, *p* = 2.2 × 10^−7^) (Figure 6A). Notably, ccfnDNA and ccfmtDNA showed similar age‐related patterns to those of miRNAs with which they correlate, ccfnDNA and *miR‐483‐5p* increasing during aging, whereas ccfmtDNA, *miR‐126‐3p*, and *miR‐21‐5p* showed no correlation with age.

We further performed a principal component analysis (PCA) and heatmap clustering analysis using most of the parameters measured in our study. Both analyses showed no separation of the samples according to age and/or sex (Figure S14). In contrast, the variable correlation plots showed two clusters: one on the lower right composed of ccfnDNA quantification by different qPCR assays and a second on the upper right including mtDNA, mRNA, and rRNA qPCR‐based quantifications, while integrity indexes had little contribution to the variance of the first two principal components and were localized in the central region (Figure 6B). Similar variable clusters were also visible in the heatmap clustering analysis (Figure S14B).

## 4 Discussion

In this study, we developed an optimized workflow for the comprehensive analysis of several types of plasma ccfNAs during aging in healthy individuals. It enabled us to perform an integrative and multiparametric analysis of different ccfNAs, including ccfDNA (nuclear and mitochondrial) and ccfRNA (mRNA, rRNA, and miRNA), to investigate the age‐related changes in ccfNA levels and integrities, as well as in ccfDNA methylation. To our knowledge, this is the first study investigating both ccfDNA and ccfRNA variations in this context.

Thus, we have reported for the first time correlation analyses between plasma ccfDNA and ccfRNA levels, as well as integrative analyses using PCA and 2D heatmap clustering of the abundance, integrity, and methylation of plasma ccfNAs (Figures 6 and S14). We showed that ccfmRNA and *18S*‐ccfrRNA levels correlated positively with ccfnDNA and ccfmtDNA levels, with the latter exhibiting the strongest correlation with both ccfRNA species. In addition, PCA and heatmap clustering analysis revealed two clearly separated variable clusters characterized by the abundance and integrity of (i) ccfmtDNA, ccfmRNA, and ccfrRNA, or (ii) ccfnDNA. This suggests that ccfmtDNA, ccfmRNA, and ccfrRNA share a similar pattern in plasma, distinct from that of ccfnDNA. Collectively, all our results highlight that total ccfDNA and ccfRNA levels in an individual's plasma are closely related, possibly through a global and common phenomenon such as the release mechanism in the blood. Thus, an increase in cellular apoptosis, necrosis, and/or secretion might potentially explain the elevated plasma levels of both ccfDNA and ccfRNA species. Furthermore, the similar pattern observed in refined analyses for ccfmtDNA and ccfRNA, but not ccfnDNA, leads us to speculate that there is also a common release, maintenance, and/or clearance mechanism into the bloodstream for these specific types of ccfNAs. For example, we could speculate that platelets, which have recently been identified as a major contributor of plasma ccfRNA (Vorperian et al. 2022), could release both RNA and mtDNA but not nDNA into the blood.

We quantified total ds ccfDNA, total linear ds ccfDNA, and ccfnDNA using the Qubit fluorometer, the Femto Pulse system, and nuclear DNA qPCR assays, respectively. These three types of ccfDNA are generally considered equivalent in most studies as most ccfDNA is in linear form and double‐stranded (Thierry et al. 2016), and due to the extremely high contribution of ccfnDNA to total ccfDNA (Meddeb et al. 2019; Panagopoulou et al. 2024). We observed a significant increase in ccfnDNA levels during aging with Qubit and qPCR assays (*Kpn I* repeat and *APP‐67* bp) quantification, respectively. Significant correlations were obtained only with the shortest *Kpn I* assays up to 168 bp (Figure 2 and Figure S4), while most of the significant differences were observed between the oldest group (50–66 y.o) and the youngest (19–39 y.o) in age‐group comparisons (Figures 2, S4 and S5). Significant differences were mainly captured by smaller qPCR assays, indicating that the inter‐group and inter‐individual variabilities of ccfDNA levels might predominantly reside in highly fragmented DNA. Our results align with those of Meddeb et al. (2019), who reported increased ccfDNA levels in plasma of healthy individuals aged 18–69 years. In addition, the increased ccfnDNA levels observed here in older women are also consistent with previous studies (Jylhava et al. 2011; Zhong et al. 2007). The increase in ccfDNA levels with age has been linked to increased inflammation, which is associated with increased cell death in the elderly (Jylhava et al. 2011; Meddeb et al. 2019). Furthermore, integrity analyses revealed a significant decrease in total ccfDNA and ccfnDNA integrity, using the Femto Pulse and *Kpn I* qPCR assays, respectively (Figures 2 and S8). The higher apoptosis‐to‐necrosis ratio in older individuals previously described (Behrens et al. 2011) may explain an increased contribution of apoptosis to ccfDNA release, resulting in smaller ccfnDNA fragments around 166 bp (Ungerer et al. 2020). Our global ccfDNA methylation results revealed no significant change with age (Figure 3), contrasting with another study that identified hypomethylation of *Line1* elements using MS‐PCR (Erichsen et al. 2018). These discrepancies could be due to the different CpGs analyzed and require further validation. Collectively, our findings suggest the potential of ccfnDNA level and integrity as a biomarker of aging.

Although not protected by a nucleosome structure, we were able to easily detect 100‐bp and 400‐bp ccfmtDNA fragments at up to 3.49 × 10^6^ and 1.91 × 10^6^ copies/mL of plasma, respectively (Figure 2), as well as very high proportions (up to 100%) and copy numbers (up to 1.2 × 10^4^) of circular mtDNA in some plasma samples (Figure S6). In contrast to ccfnDNA, ccfmtDNA showed no significant age‐related variations (Figure 2), which is consistent with some studies reporting no age‐related changes in ccfmtDNA levels (Jylhava et al. 2013; Meddeb et al. 2019). However, other studies described a significant increase in ccfmtDNA levels with age, either among healthy participants aged 20–69 years (Padilla‐Sanchez et al. 2020) or aged from 1 to 104 years with a peak value at 90 (Pinti et al. 2014). Interestingly, we also observed a highly variable number of ccfmtDNA copies per genome equivalent among participants (RSD = 240.5%), with a clear but non‐significant decrease with age for both total and circular ccfmtDNA (Figures 2 and S6). Such variability has also been reported in another study (Meddeb et al. 2019) and might potentially be related to the forms of cell‐free mtDNA in blood, which could be free (Bisserier et al. 2021; Szilagyi et al. 2020), in an intact form in circulating mitochondria (Al Amir Dache et al. 2020; Szilagyi et al. 2020), or encapsulated in circulating vesicles (Al Amir Dache et al. 2020; Bisserier et al. 2021; Szilagyi et al. 2020). It could also be related to the particular status of an individual. For example, the inflammatory status was shown to correlate with ccfmtDNA level (Pinti et al. 2014). It could also be related to release, maintenance, and/or degradation mechanisms, which could be distinct from those of ccfnDNA. The detection of large ccfmtDNA fragments (400 bp) and of the circular form at very high genome equivalent levels in some individuals, as well as the positive correlation between the proportions of circular ccfmtDNA and total ccfmtDNA levels, support the hypothesis of protective mechanisms (Al Amir Dache et al. 2020).

Little was known about ccfRNA variations in plasma samples of healthy individuals, and here we report for the first time a significant age‐related increase of both ccfmRNA and ccfrRNA levels (Figure 4), similar to ccfnDNA. Quantities measured with *GAPDH* and *18S* qPCR assays showed a positive correlation with age, and higher ccfRNA levels in the oldest participants compared to the youngest. Older women also had higher plasma ccfmRNA levels compared to younger women, as was found with ccfnDNA. Given the global deterioration of transcriptomic activity (Gupta et al. 2021; Perez‐Gomez et al. 2020) and decrease in ribosome abundance with aging (Anisimova et al. 2018, 2020), this increase might be potentially caused by an increase in the release mechanism. Unlike ccfnDNA, we did not observe any age‐related variation in *18S* rRNA integrity. In addition, we observed a high proportion of the large 270‐bp rRNA fragment compared to the small 115‐bp and 59‐bp fragments, and also a high inter‐individual variability of *18S* rRNA, *GAPDH*, and *B2M* mRNA copies in plasma (Figure 4). We speculate that the maintenance of relatively large ccfrRNA fragments in blood might be due to a protective mechanism or structure. However, we cannot ensure that this integrity also includes ccfmRNAs, although they have been shown to be protected in EVs (Kim et al. 2023). Moreover, we could also speculate that the high variability in ccfrRNA and ccfmRNA levels among individuals could be caused by different release, maintenance, and/or clearance mechanisms. Despite the lack of attention given to ccfmRNA and ccfrRNA in ccfNA research, they might display a potential as plasma biomarkers of aging.

Among the candidate age‐related miRNAs analyzed by RT‐qPCR, only *miR‐483‐5p* exhibited a positive correlation with age, with significantly higher levels in the two oldest groups and in the oldest men compared to Group 1 and the youngest men, respectively (Figure 5). This result was also observed in miRNA‐sequencing analysis of 24 additional plasma samples (Figure S13 and Table S2). Our results are consistent with a previous study (Rusanova et al. 2018), where higher levels of *miR‐483* were found in aged individuals (mean age = 76.6 y.o) compared to young subjects (mean age = 20.5 y.o). *miR‐483‐5p* also correlated with the pro‐inflammatory cytokine IL‐8 in the same study, while it is also related to the melatonin synthesis pathway (Clokie et al. 2012), and could therefore be implicated in the melatonin decay observed in aging (Martin Gimenez et al. 2022; Rusanova et al. 2018). Therefore, *miR‐483‐5p* could both be a potential biomarker of inflammation and of healthy aging, as melatonin was described as a hormone with anti‐aging properties (Martin Gimenez et al. 2022). In contrast to previous studies (Accardi et al. 2022; Ameling et al. 2015; Olivieri et al. 2014, 2012), surprisingly, we did not observe significant changes in *miR‐21‐5p* and *miR‐126‐3p* levels during aging in both RT‐qPCR and miRNA‐sequencing experiments. However, the design of these studies included oldest participants, that is, octogenarians and nonagenarians, which could explain these discrepancies. Our miRNA‐sequencing experiments also identified several miRNAs associated with aging, including *miR‐23b‐5p*, *miR‐30c‐5p*, and *miR‐30b‐5p*, which were previously reported as age‐associated ccfmiRNAs (Ameling et al. 2015). These associations with aging will require further validation as most of them lost significance after the FDR correction.

While our results provide novel insights into age‐related changes in both ccfDNA and ccfRNA levels, their high inter‐individual variability observed in our cohort of healthy individuals—also reported for ccfDNA in previous studies (Barbany et al. 2019; Meddeb et al. 2019)—may limit their applicability as biomarkers of aging and/or healthy aging. However, this limitation could be mitigated by different strategies, including the use of (i) optimal (pre‐)analytical conditions, (ii) combined biomarkers, and (iii) longitudinal samples from the same individuals. Thus, some studies using lower centrifugation speeds for plasma isolation (700 g and 1,000 g) and quantifying directly total ccfDNA in plasma in some analyses showed lower inter‐individual variability while maintaining significant age‐related differences (Jylhava et al. 2012, 2013). Moreover, a composite biomarker could be developed by combining absolute ccfDNA and ccfRNA concentrations with other parameters showing less inter‐individual variations but remaining significantly associated with aging, such as ccfDNA integrity indexes (Figure 2D). Additionally, absolute ccfDNA and ccfRNA concentrations could be used in longitudinal plasma samples from the same individuals to detect changes over time and aging that could be indicative of health conditions (Tosevska et al. 2016). Further studies are needed to evaluate the potential of absolute ccfDNA and ccfRNA concentrations as biomarkers of aging and/or healthy aging.

Finally, we would like to highlight some limitations of our study. Although larger than most similar studies, the cohort (*n* = 139) and group sizes remain limited, especially when considering sex and age together. The absence of individuals over 66 years of age may have prevented the identification of age‐related changes sometimes observed in other studies (Accardi et al. 2022; Ameling et al. 2015; Olivieri et al. 2014, 2012). Furthermore, as our study was mainly based on PCR analysis targeting specific sequences, we did not fully exploit the information contained in ccfNAs, such as the nucleosomal footprint present in ccfDNA. A deeper investigation of the genome, epigenome, and transcriptome by sequencing would provide a more comprehensive understanding of ccfNA variations during aging and would also enable multi‐omic analyses. Another limitation of our study concerns the analysis of ccfNAs without considering their specific origin, particularly those enclosed in actively secreted EVs. The analysis of ccfNAs contained in EVs would have been important, as they play a role in intercellular communication and can carry information about the healthy or pathological state of the donor cells, tissues, and/or organs (De Toro et al. 2015; Mathieu et al. 2019).

In conclusion, our optimized multiparametric analysis workflow allowed highlighting numerous ccfNA variations occurring during aging and identifying potential biomarkers of aging. It enables a better understanding of ccfNA biology in healthy individuals during aging, which will also improve their use in pathological contexts.

## Author Contributions

N.P.T., L.M.H., A.D., C.D., I.H., and C.H. performed the experiments. N.P.T., A.D., M.S., and A.H.‐K. analyzed the data, made the figures and tables. A.H.‐K. conceived and supervised the project. N.P.T. and A.H.‐K. drafted the first version of the manuscript. All authors read, edited, and approved the final version of the submitted manuscript.

## Conflicts of Interest

The authors declare no conflicts of interest.

## Supporting information


Data S1.

## Data Availability

The data that support the findings of this study are available from the corresponding author upon reasonable request.
