# Supplementary material for: Integrative Multiparametric Analysis of Circulating Cell‐Free Nucleic Acids of Plasma in Healthy Individuals During Aging
Source: Aging Cell. 2025 Jun 19;24(9):e70133. doi: 10.1111/acel.70133 (PMC12419850; doi:10.1111/acel.70133)
Supplement: Supplementary file 1 — Data S1. [file ACEL-24-e70133-s001.pdf]

Supplementary data for: Integrative multiparametric analysis of circulating cell-free nucleic acids of plasma in healthy individuals during aging

Nicolas P. Tessier[1], Lise M. Hardy[1], Florence Mauger[2], Antoine Daunay[1], Christian Daviaud[2], Ilef Hchaichi[1], Caroline Horgues[2], Mourad Sahbatou[1], Hélène Le Buanec[3], Jean-François Deleuze[1,2] & Alexandre How-Kit[1‡]

[1] Laboratory for Genomics, Foundation Jean Dausset – CEPH, Paris, France

[2] Université Paris-Saclay, Commissariat à l'Energie Atomique et aux Energies Alternatives, Centre National de Recherche en Génomique Humaine, Evry, 91057, France.

[3] Saint-Louis Research Institute, INSERM U976 - HIPI Unit, University of Paris, Paris, France

[‡] ***Correspondence to:***

Alexandre How-Kit, Ph.D., Laboratory for Genomics, Foundation Jean Dausset - CEPH, Paris, F-75010, France, Tel.: +33-(0)1- 53725146, email: alexandre.how-kit@fjd-ceph.org

Keywords

Plasma, circulating cell-free nucleic acids, circulating cell-free DNA, circulating cell-free RNA, aging, DNA methylation, miRNA

Supplementary Figures

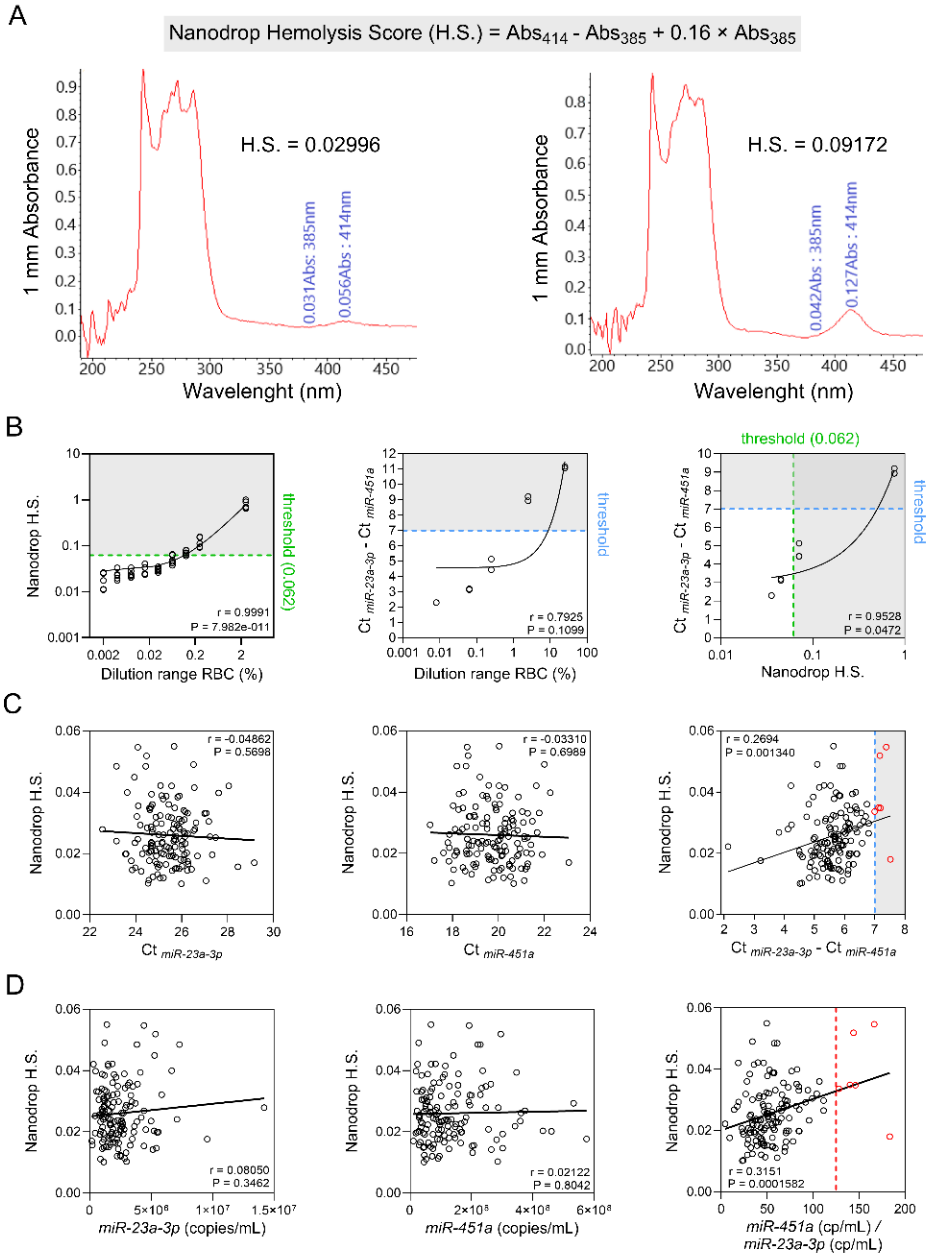

Figure S1. Hemolysis assessment of plasma samples by spectrophotometry and qPCR (n = 139 samples). A, Nanodrop H.S. formula using the plasma absorbance measured at 385 nm and 414 nm (Appierto et al., 2014). Examples of two absorbance profiles obtained with artifically hemolyzed control plasma samples spiked with 0.25 % (left) and 0.031 % (right) of red blood cells (RBC). **B,** H.S. and $Ct_{\mathit{mir\text{-}23a\text{-}3p}} - Ct_{\mathit{miR\text{-}451a}}$ obtained with Nanodrop (left) and qPCR (middle), respectively, using control plasma samples spiked with different percentages of RBC. The scores obtained with both methods on control samples are shown on the left . **C,** Correlation of Nanodrop H.S. with the Ct values of the two control miRNAs used for hemolysis assessment (*miR-23a-3p* (left) and *miR-451a* (middle)), and with the calculated qPCR-based hemolysis score ($Ct_{\mathit{miR\text{-}23a\text{-}3p}} - Ct_{\mathit{miR\text{-}451a}}$) obtained for plasma samples of the study cohort (n = 139 samples). Red circles in the grey area (right) indicate hemolyzed samples with $Ct_{\mathit{mir\text{-}23a\text{-}3p}} - Ct_{\mathit{miR\text{-}451a}} > 7$. **D,** Correlation of Nanodrop H.S. with absolute concentrations of control miRNAs (miR-23a-3p (left) and miR-451a (middle)), and with the ratio of *miR-451a* to *miR-23a-3p* obtained for plasma samples of the study cohort (n = 139 samples). Red circles indicate hemolyzed samples with $Ct_{\mathit{mir\text{-}23a\text{-}3p}} - Ct_{\mathit{miR\text{-}451a}} > 7$. Pearson's *r* correlation coefficients and associated *p*-values are indicated in each graph. *p*-values < 0.05 are considered significant. Dashed lines represent the score thresholds and the grey zone corresponds to hemolyzed plasma samples in **B**-**D**. Linear regression lines are shown in black in **B**-**D**.

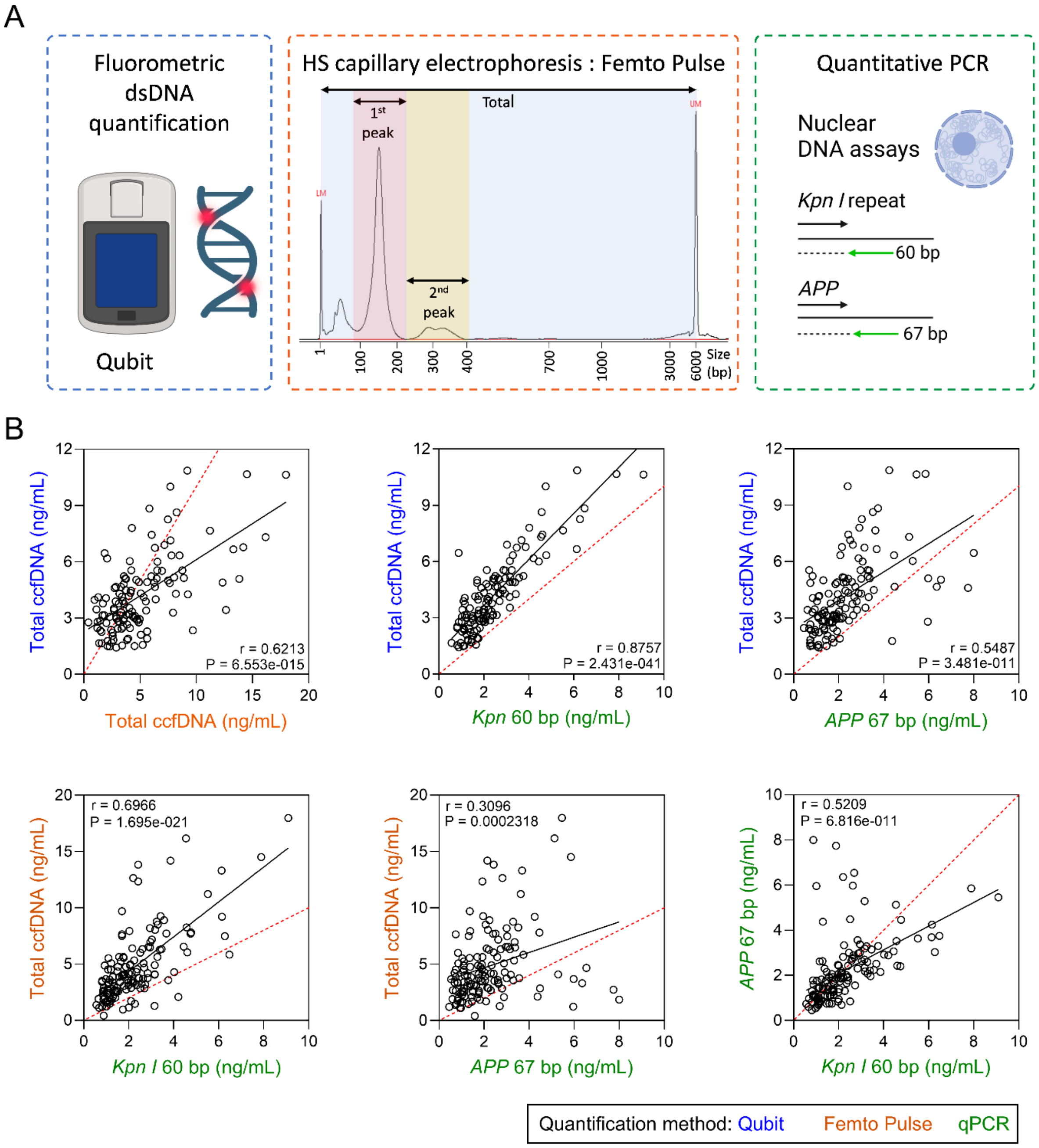

Figure S2. Comparison of three plasma ccfDNA quantification methods (n = 139). A, Quantification methods used: fluorometry, high-sensitivity capillary electrophoresis and quantitative PCR. **B,** Correlation analysis of ccfDNA quantification by the three methods used. Linear regression lines are shown in black and *Y* = *X* lines are red and dashed. Pearson's *r* correlation coefficients and associated *p*-values are given in each graph. *p*-values < 0.05 are considered significant.

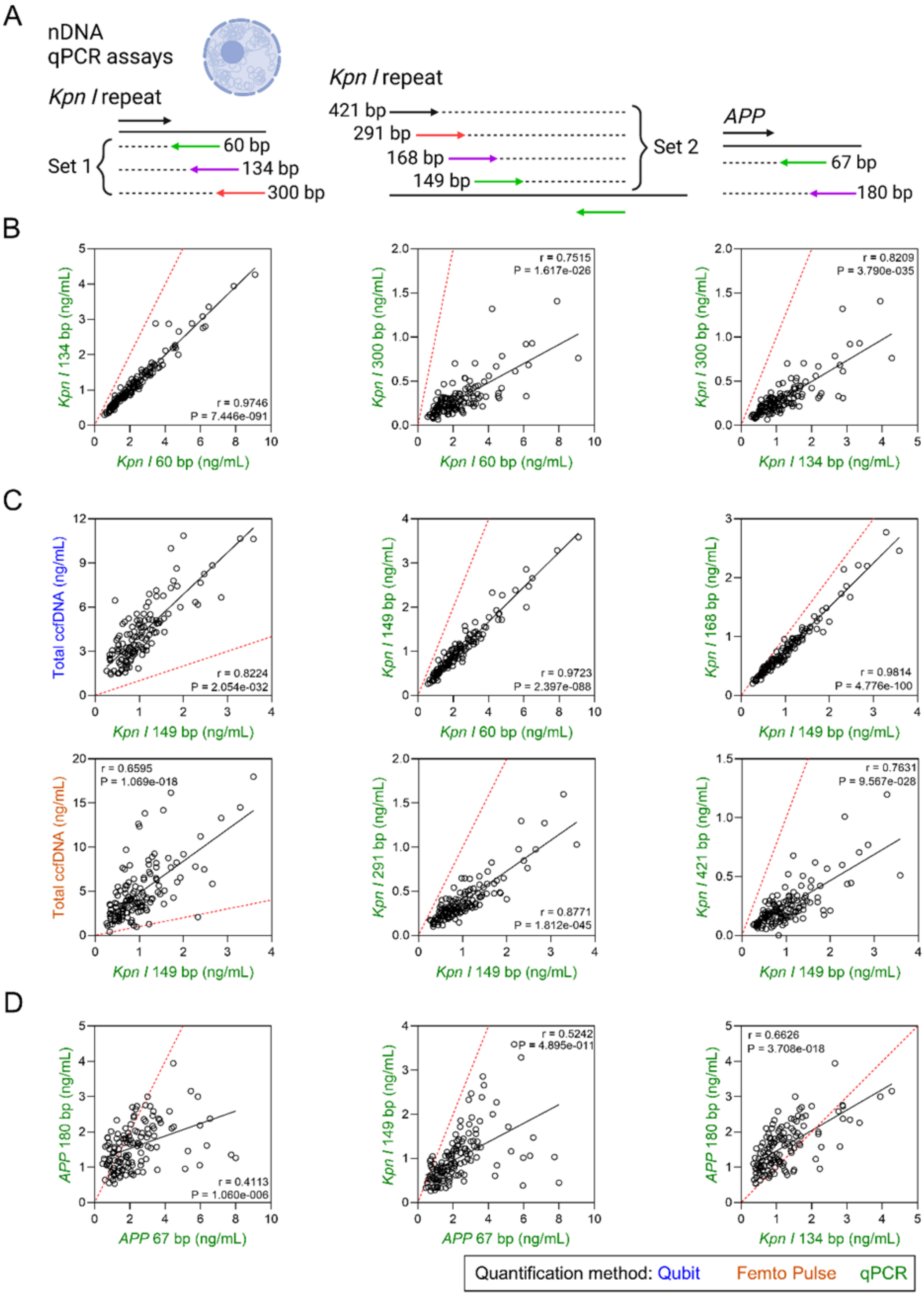

Figure S3. Correlation analysis of ccfDNA quantification methods (n = 139 samples). A, qPCR assays used for ccfDNA quantification**. B,** Correlation analyses of ccfDNA quantifications based on the set 1 of *Kpn I* repeat qPCR assays. **C,** Correlation analyses of ccfDNA quantifications using the set 2 of *Kpn I* repeat qPCR assays, the Qubit and the Femto Pulse. **D,** Correlation analyses of ccfDNA quantifications using the *APP* assays and the set 2 of *Kpn I* repeat assays. Linear regression lines are shown in black and *Y* = *X* lines are red and dashed. Pearson's *r* correlation coefficients and associated *p*-values are indicated in each graph. *p*-values < 0.05 are considered significant.

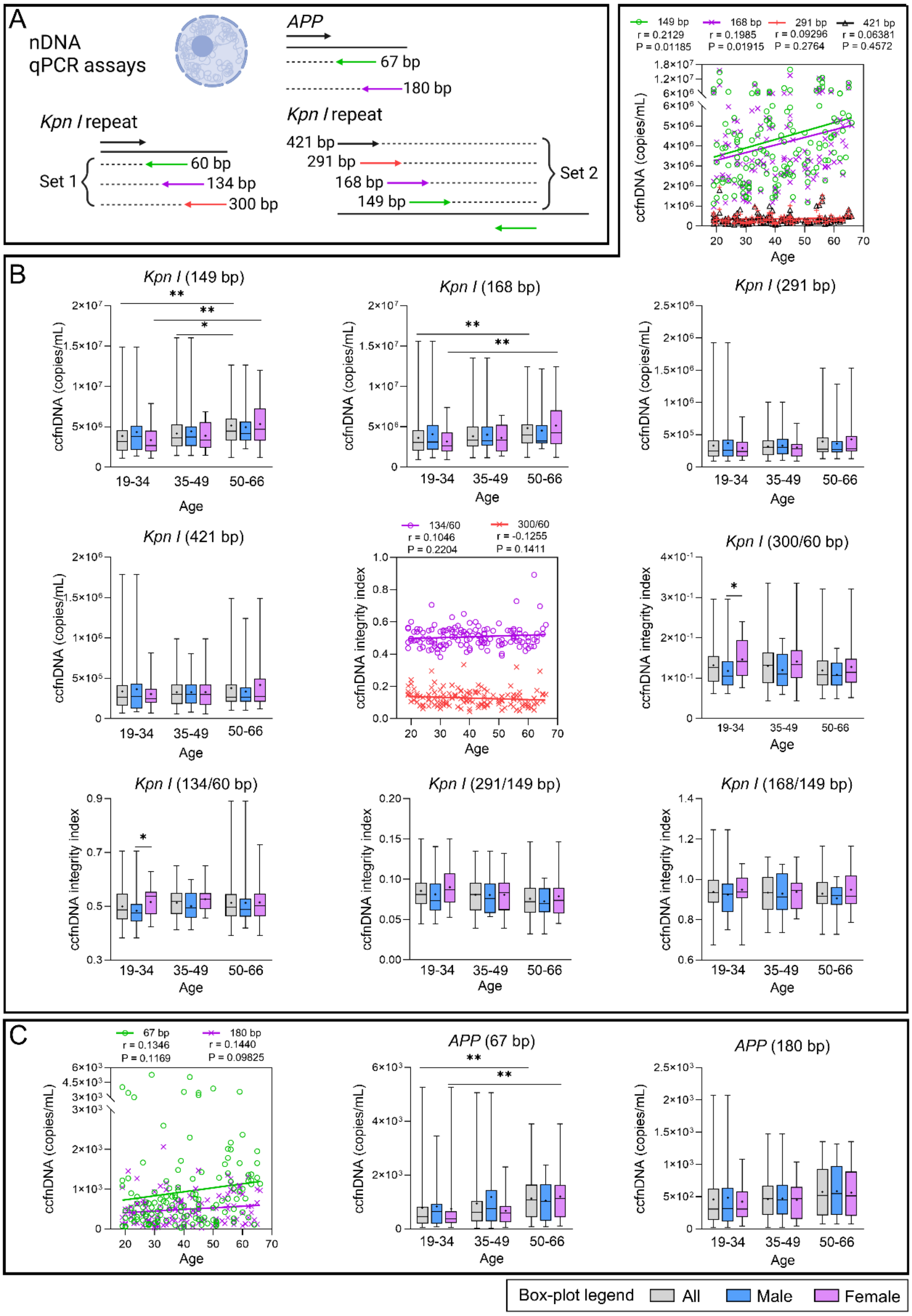

Figure S4. ccfDNA quantity and integrity analysis during aging using *Kpn I* and *APP* qPCR assays (n = 139 samples). A, qPCR assays used for ccfnDNA analysis. **B,** ccfnDNA quantity and integrity analyses using both sets of *Kpn I* repeat assays. The integrity index is calculated as the ratio of the *Kpn I* repeat copy numbers obtained from the larger amplicons (300 and 134 bp for set 1, 291 and 168 bp for set 2) to the smallest one (60 and 149 bp respectively). **c,** ccfnDNA quantity analysis using the *APP* qPCR assays. Mann-Whitney U tests were performed in box-plots between each age group and between men and women of a same age group. The '+' symbol indicates the mean value obtained for each group. Pearson's *r* coefficients and associated *p*-values as well as linear regression lines are indicated in scatterplots. *p*-values < 0.05 are considered significant; * < 0.05, ** < 0.01.

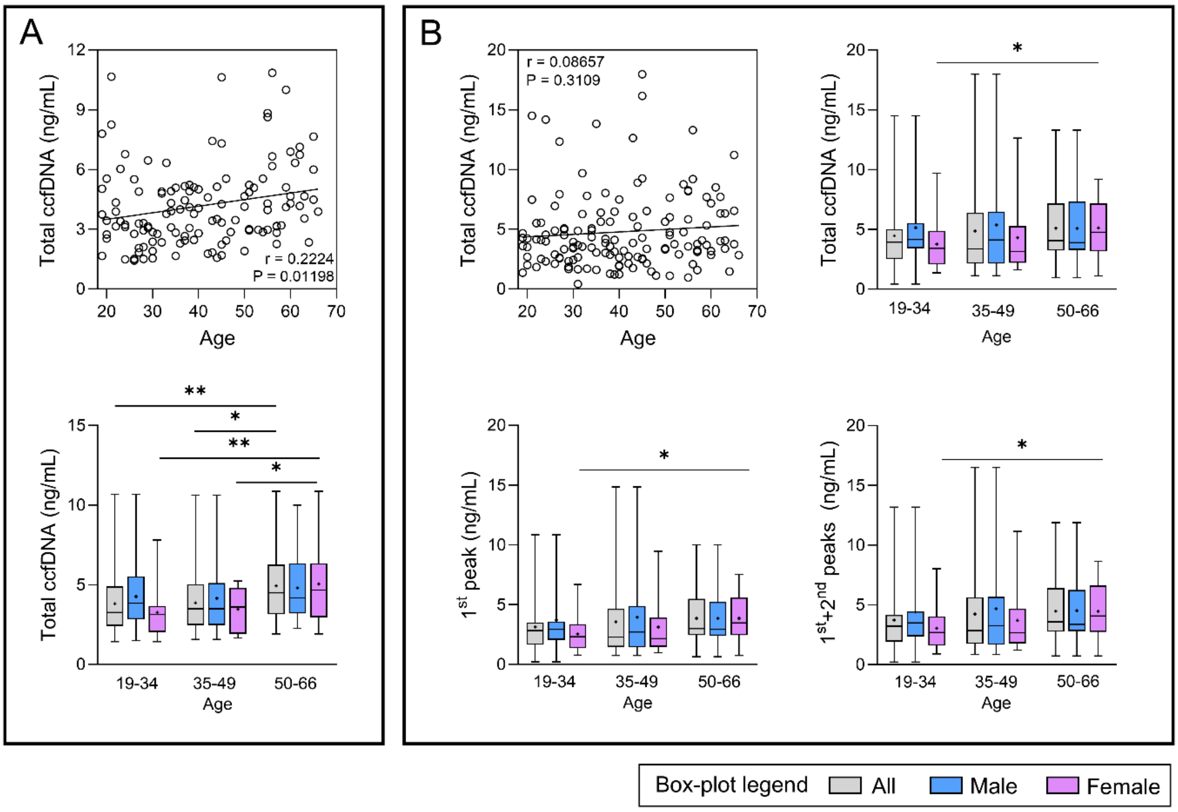

Figure S5. Variation of total ccfDNA concentrations during aging using the Qubit and the Femto Pulse (n = 139 samples). A, Total ccfDNA amount obtained by fluorometric quantification using Qubit. **B,** Quantification of total ccfDNA by capillary electrophoresis using the Femto Pulse. Concentrations are measured on the Femto Pulse ccfDNA profile considering the 1st peak, the 1st and 2nd peak, and the total concentration. Mann-Whitney U tests were performed in box-plots between each age group and between men and women of the same age groupThe '+' symbol indicates the mean value obtained for each group. Pearson's *r* coefficients and associated *p*-values as well as linear regression lines are indicated in scatterplots. *p*-values < 0.05 are considered significant; * < 0.05, ** < 0.01.

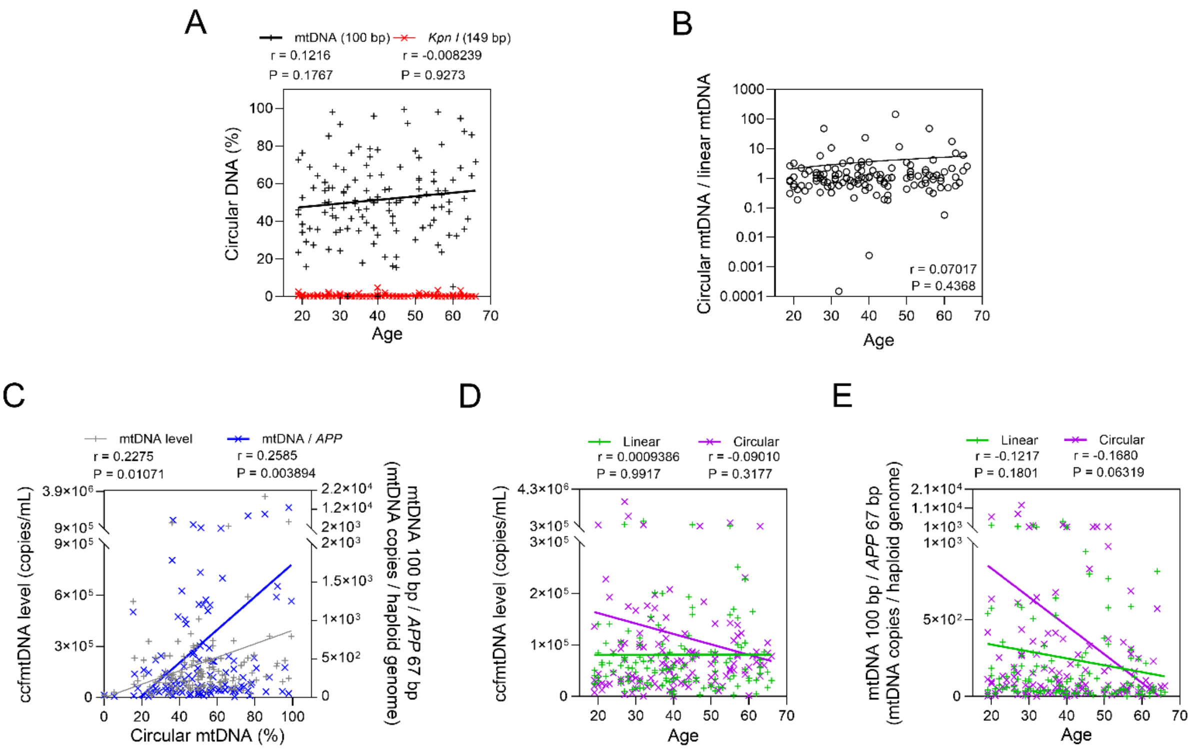

Figure S6. Assessment of the linear and circular forms of ccfmtDNA in plasma samples during aging (n = 125). A, Variation of the percentage of circular mtDNA during aging. The percentage of circular *Kpn I* allows to estimate the efficiency of Exonuclease V digestion. **B,** Ratios of circular to linear mtDNA during aging. **C,** Correlation analyses between the percentage of circular mtDNA and total ccf-mtDNA levels, as well as total ccf-mtDNA copy number per genome equivalent. **D,** Variation in circular and linear ccfmtDNA levels with age. **E,** Variation in circular and linear ccfmtDNA copy number per genome equivalent with age. Pearson's *r* coefficients and associated *p*-values as well as linear regression lines are shown. For these experiments, 1 µL of ccfNAs was treated using 1U of Exonuclease V (NEB) at 37°C during 30 min, followed by enzyme inactivation at 70°C during 30 min, in 1X NEBuffer™ 4, in a final reaction volume of 10 µL. For each sample, a parallel reaction without the Exonuclease V was also performed under the same conditions. 2 µl of each reaction were used as template for qPCR analysis using mtDNA 100-bp and *Kpn I* 149-bp assays (see Methods). Digestion efficiency was measured using the *Kpn I* assay $(2^{-Ct\ Exo\ V^-} - 2^{-Ct\ Exo\ V^+})/(2^{-Ct\ Exo\ V^-})$ and showed a mean value of 99.52% ± 0.72 (min = 95.16%, max = 99.97%). The fraction of circular DNA in a sample was calculated using the formula: $2^{-Ct\ Exo\ V^+}/2^{-Ct\ Exo\ V^-}$. Efficiencies of both assays were not affected by the enzyme digestion mix and were close to 2. Absolute concentrations of circular and linear ccfmtDNA were obtained by applying the fraction of each form to the total ccfmtDNA levels. Exo V: Exonuclease V.

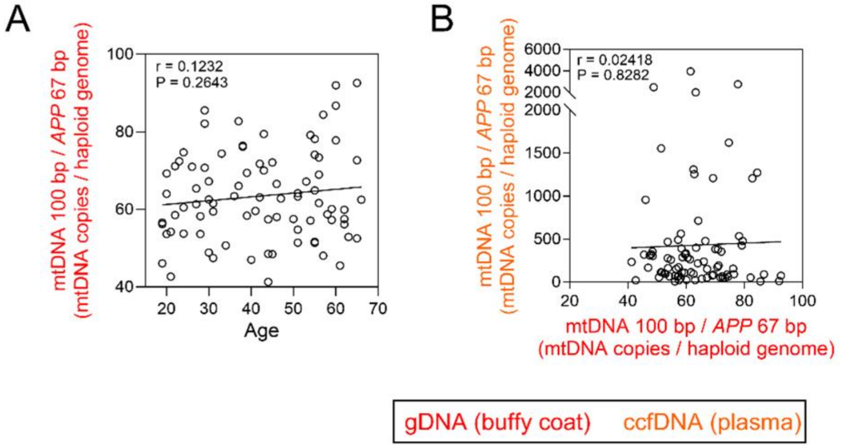

Figure S7. Correlation of mtDNA copies per genome equivalent in buffy coats with age and with plasma values (n = 84). A, Variation in buffy coat mtDNA copies per genome equivalent during aging. **B,** Correlation of plasma and buffy coat mtDNA copies per genome equivalent. Buffy coats were isolated in parallel with plasma isolation, after the first centrifugation of blood at 1 600 g, and stored at -80°C before DNA extraction. DNA was extracted from 100 µL of buffy coat samples using the QIAmp DNA blood mini Kit (Qiagen) according to the manufacturer's instructions. DNA quantification was performed using the Qubit^{TM} dsDNA HS assay Kit on a Qubit 3 Fluorometer (Thermo Fisher Scientific) according to the manufacturer's instructions. Absolute quantification of mtDNA (100-bp assay) and *APP* (67-bp assay) copy numbers was performed by qPCR under the same experimental conditions described in the Methods section, using 10 ng of DNA per PCR reaction as template.

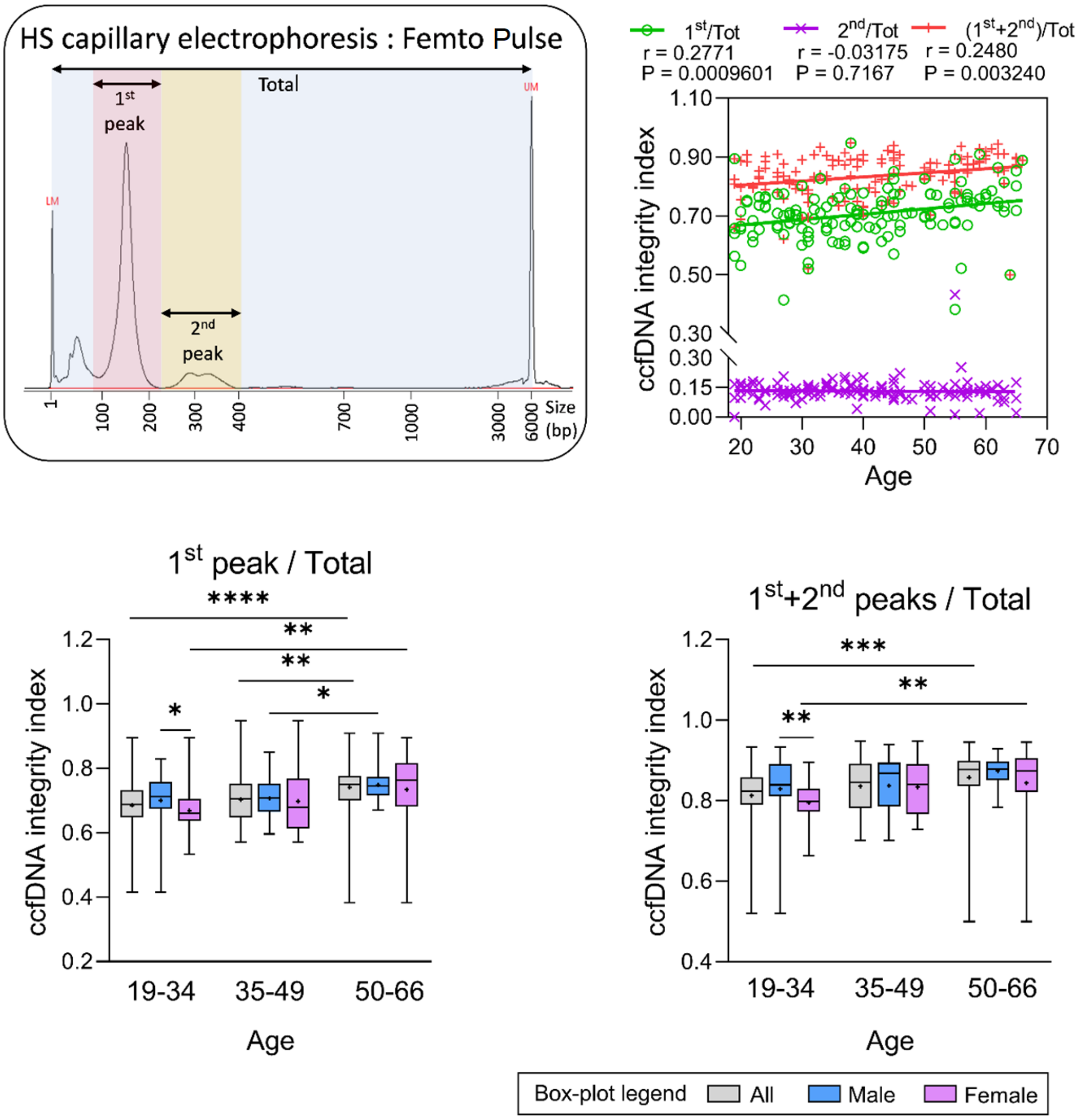

Figure S8. Integrity analysis of total ccfDNA using the Femto Pulse capillary electrophoresis (n = 139 samples). Example of a ccfDNA profile obtained on the Femto Pulse system (top-left) with the first two ccfDNA peaks. The integrity index is calculated as the ratio of the 1st and/or 2nd peak concentration to the total ccfDNA concentration. Mann-Whitney U tests were performed in box-plots between each age group and between men and women of the same age group. The '+' symbol indicates the mean value obtained for each group. Pearson's *r* coefficients and associated *p*-values as well as linear regression lines are indicated in scatterplots. *p*-values < 0.05 are considered significant; * < 0.05, ** < 0.01, *** < 0.001, **** < 0.0001.

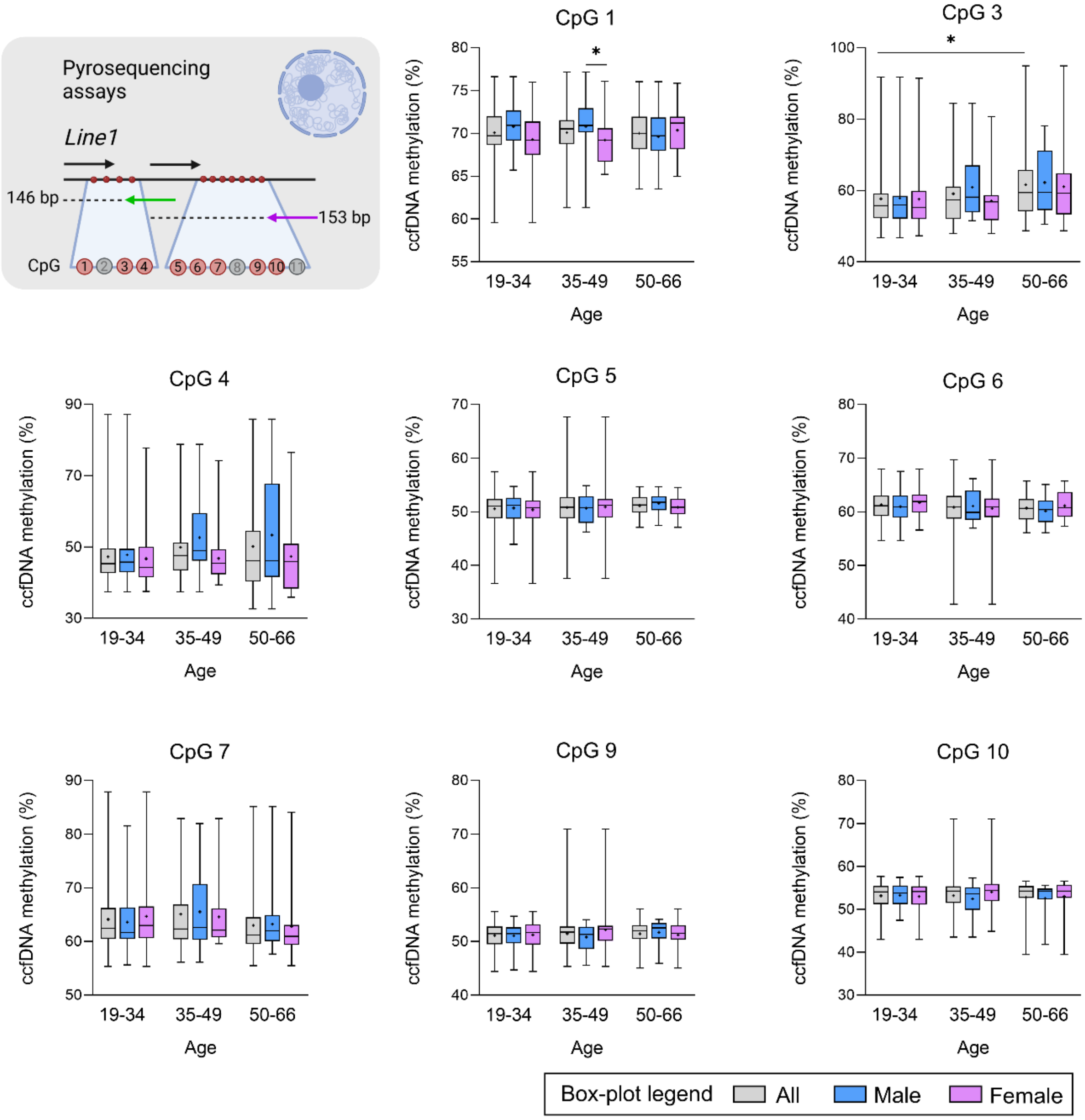

Figure S9. *Line1* ccfDNA methylation variation during aging obtained for CpG$_{1,3\text{-}7,\,9\text{-}10}$ by pyrosequencing (n = 139 samples). *Line1* pyroquencing assays used and the analyzed CpGs are indicated at the top-left. 3 CpGs were excluded for detailed analysis (shaded in grey). Mann-Whitney U tests were performed in box-plots between each age group and between men and women of the same age group. The '+' symbol indicates the mean value obtained for each group. *p*-values < 0.05 are considered significant; * < 0.05.

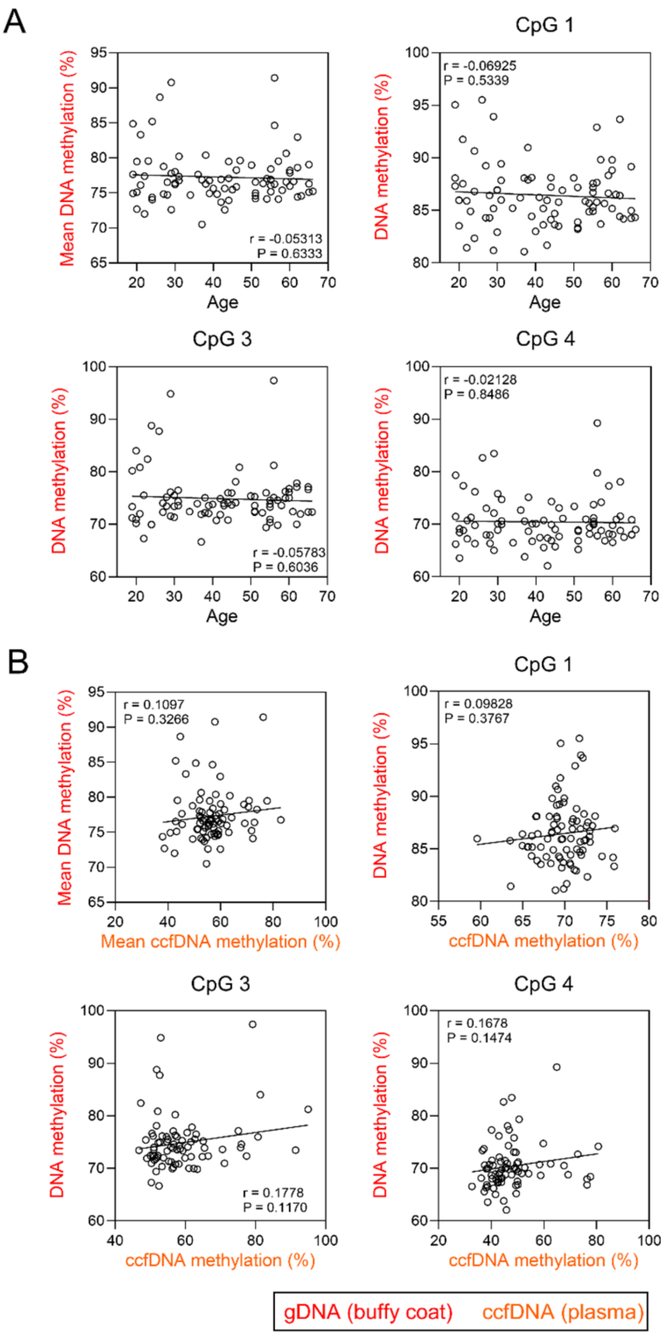

Figure S10. Correlation of *Line1* DNA methylation in buffy coats with age and plasma values (n = 84). A, Variation in *Line1* DNA methylation in buffy coat samples during aging. **B,** Correlation of *Line1* DNA methylation between plasma and buffy coat samples. DNA was extracted from buffy coat samples as described in Figure S7. Bisulfite conversion was performed on 800 ng of genomic DNA using the EpiTect Fast 96 DNA Bisulfite Kit (Qiagen) according to the manufacturer's instructions, and an elution volume of 50 µL. PCR was performed on 10 ng of converted DNA using the *Line1* 146-bp assay under the same experimental conditions described in the Methods section. Pyrosequencing analysis was performed as described in the Methods section. Only CpG_1, CpG_3, and CpG_4 were included in our DNA methylation analysis.

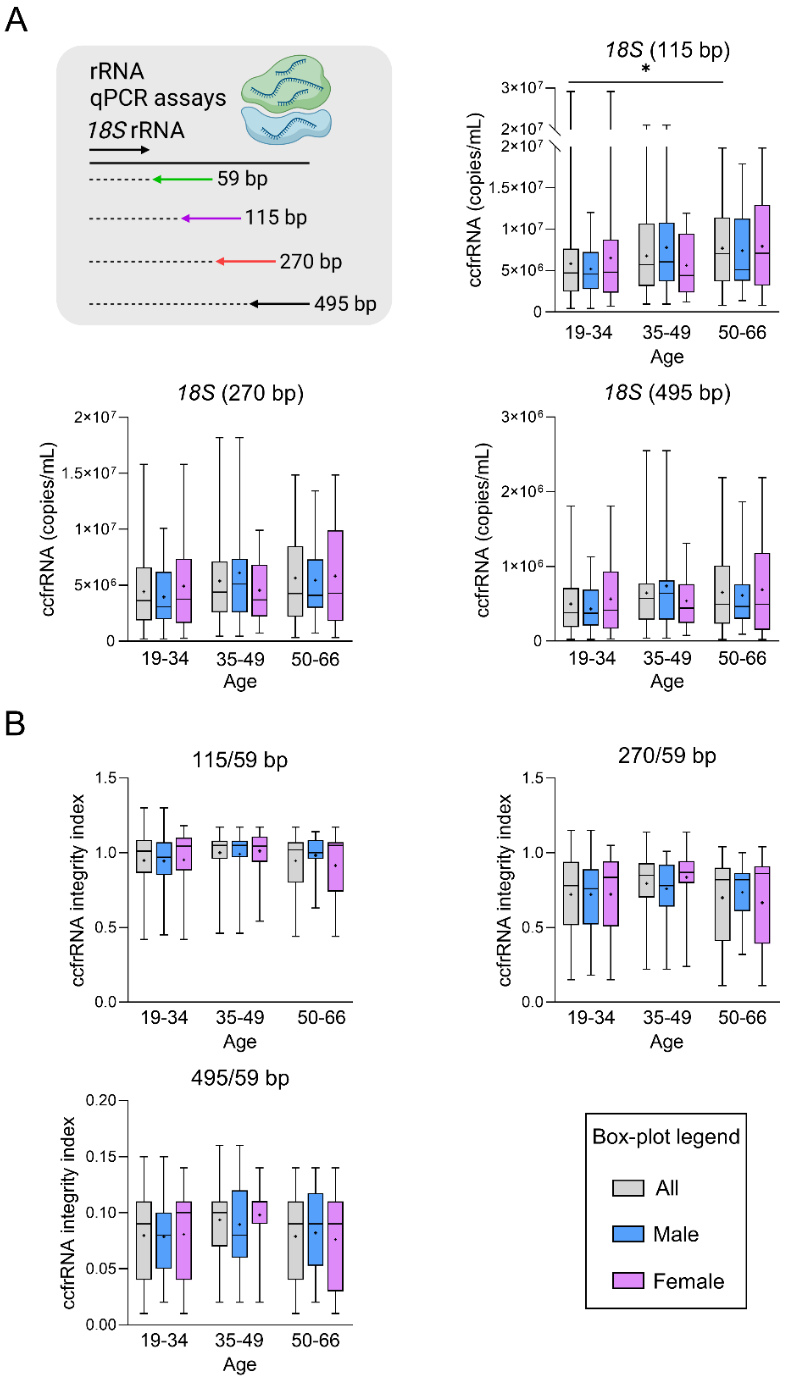

Figure S11. Variation of plasma ccfrRNA quantity and integrity during aging (n = 139 samples). A, Boxplots of *18S* ccfrRNA quantification variation during aging. *18S* qPCR assays are shown at the top-left. **B,** Assessment of ccfrRNA integrity using the integrity index, calculated as the ratio of the copy number of a larger *18S* qPCR assay amplicon to the smallest amplicon. Mann-Whitney U tests were performed in box-plots between each age group and between men and women of the same age group. The '+' symbol indicates the mean value obtained for each group. *p*-values < 0.05 are considered significant; * < 0.05.

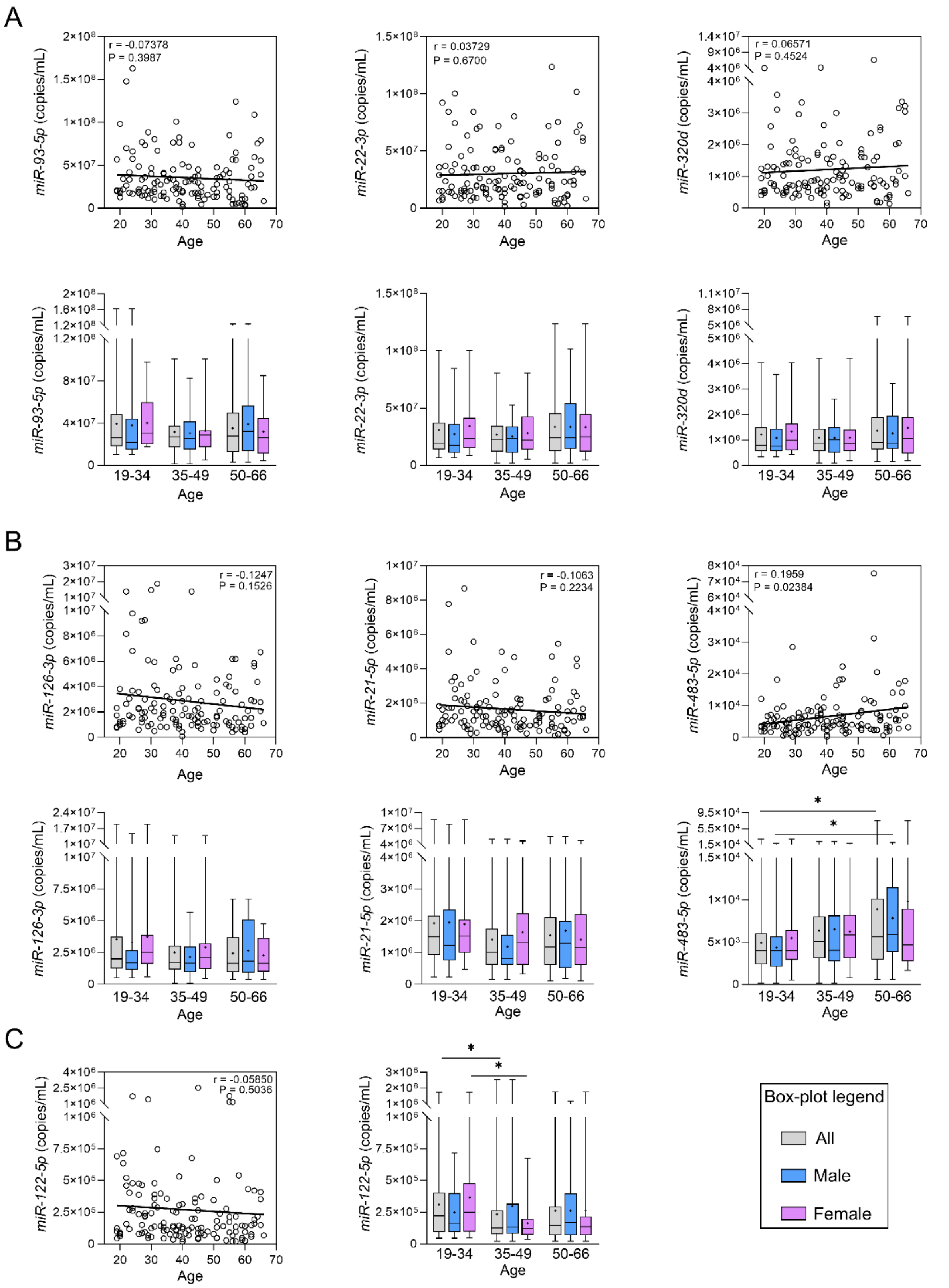

Figure S12. Variation in the copy number of different plasma ccfmiRNAs during aging (n = 133 samples). (**A**) Absolute quantification of housekeeping-miRNAs (*miR-93-5p*, *miR-22-3p*, *miR-320d*), (**B**) age-associated ccfmiRNAs (*miR-126-3p*, *miR-21-5p*, *miR-483-5p*) and (**C**) liver-specific ccfmiRNA (*miR-122-5p*) during aging. Mann-Whitney U tests were performed in box-plots between each age group and between men and women of the same age group. The '+' symbol indicates the mean value obtained for each group. Pearson's *r* coefficients and associated *p*-values as well as linear regression lines are indicated in scatterplots. *p*-values < 0.05 are considered significant; * < 0.05.

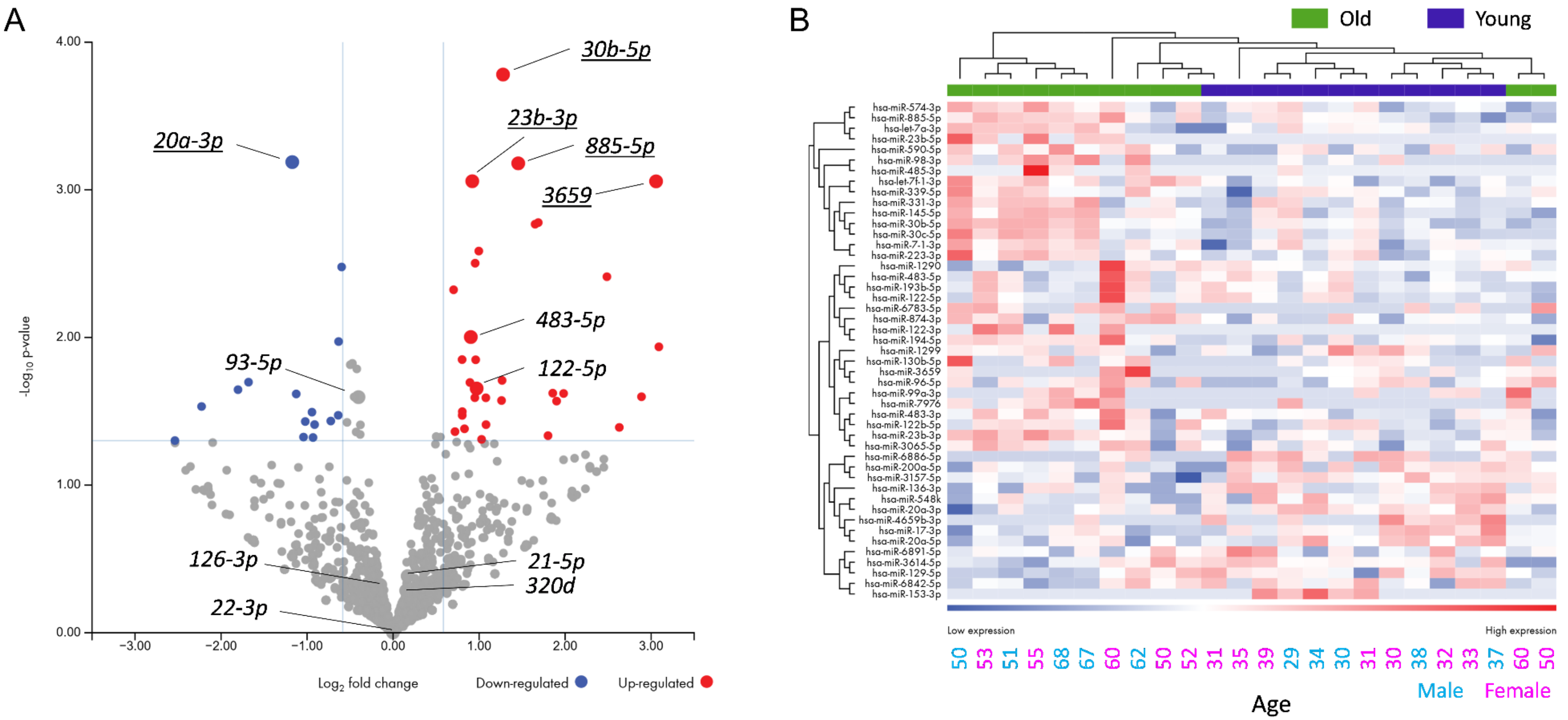

Figure S13. Differential expression analysis of miRNA-sequencing data (n = 24 samples) comparing older individuals (50-68 years old) to younger individuals (29-39 years old). A, Volcano plot showing the differential miRNA expression between the two age groups. Each dot represents a miRNA. miRNAs significantly overexpressed and underexpressed in the older group are displayed in red and in blue, respectively. miRNAs were considered differentially expressed using a p-value ≤ 0.05 (horizontal blue line) and a fold change $\leq 1/1.5$ or ≥ 1.5 (vertical blue lines). miRNAs with a FDR-adjusted p-value ≤ 0.1 are underlined. **B,** Bi-directional clustered heatmap of the differentially expressed miRNAs, identified using the same p-value and fold change thresholds. For the miRNA-sequencing experiments, miRNAs were isolated from 300 µL of plasma using the Quick-cfRNA™ Serum & Plasma Kit (Ozyme) according to the manufacturer's instructions. Plasma hemolysis was evaluated by qPCR using *miR-451a* and *miR-23a-3p* Ct values. Library preparation and sequencing were performed by the iGenSeq Platform of the Institut du Cerveau (ICM), using the QIAseq miRNA Library Kit (Qiagen) for library preparation, and single-end sequencing (20 million reads per sample) on a NovaSeq X (Illumina). Differential expression analysis was performed with GenGlobe online tool (Qiagen) using the generated FastQ files.

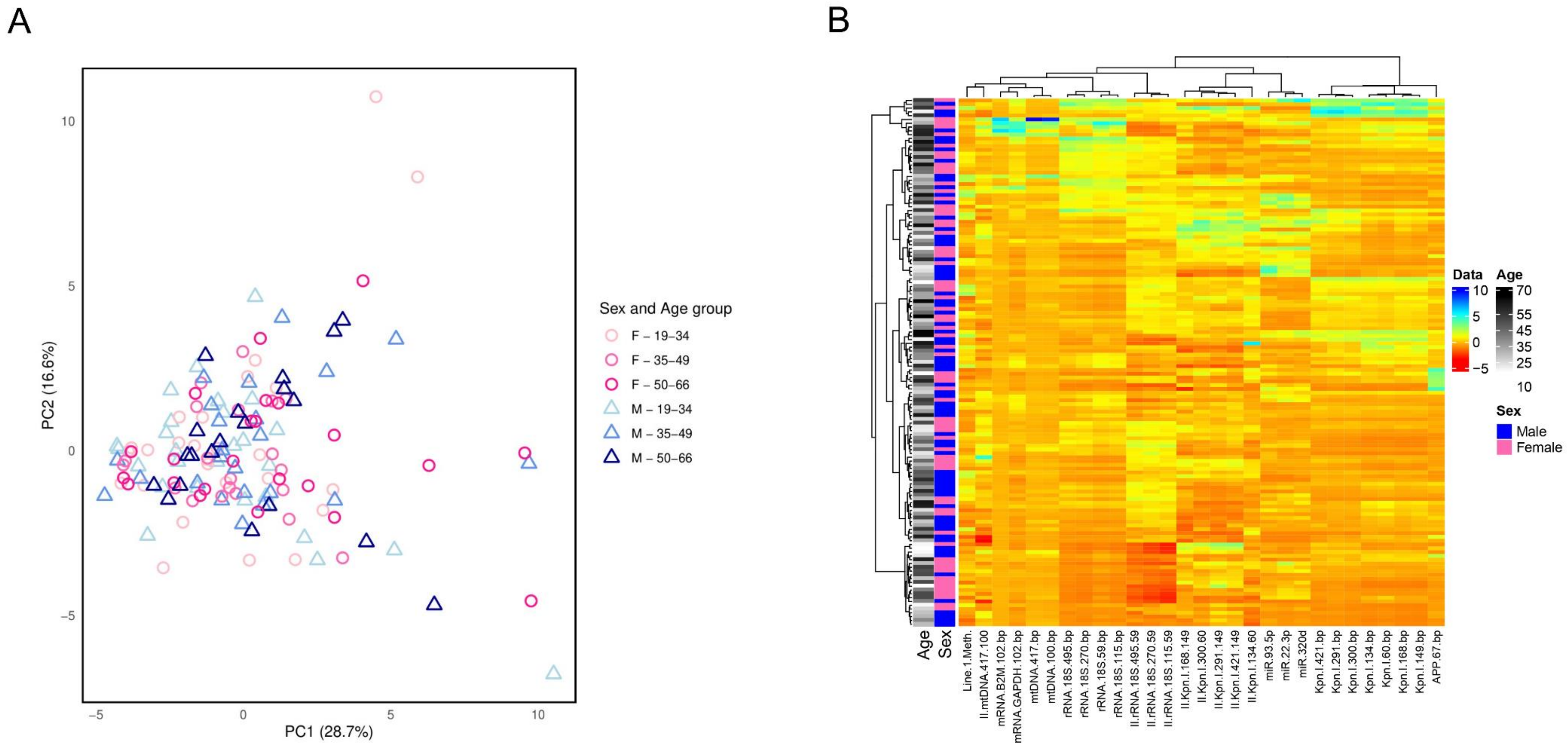

Figure S14. PCA and clustering analysis of all plasma samples (n = 139) based on ccfNA quantity, integrity and methylation data. A, PCA plot on individuals showing the contribution of the first two principal components (PC) on the samples and the variance between age and sex groups. **B,** Heatmap obtained by performing the Ward clustering method in order to find compact and spherical clusters between variables and between individuals. Before PCA and heatmap analysis, a preprocessing step called A-score was performed to scale the data by standardization. II: integrity index.

Supplementary Tables

Table S1. List of all PCR primer and probe sequences used in the study

Analysis	Assay name	Forward primer	Sequence (5' to 3')	Reverse primer	Sequence (5' to 3')	Sequencing primer/ Hydrolysis probes	Sequence (5' to 3')	Amp . size (bp)	Efficiency
**ccfnDNA**	***Kpn_I_60***	KPN_I_60_F	ATGGCTGGGTCAAATGGTATTTCC	KPN_I_60_R	GAAGTCAGTGTGGCAATTCCTCAG			60	1.99
	Kpn_I_134	KPN_I_60_F	ATGGCTGGGTCAAATGGTATTTCC	KPN_I_134_R	TGCTGGAGAGGATGGGAGAA			134	1.94
	Kpn_I_300	KPN_I_60_F	ATGGCTGGGTCAAATGGTATTTCC	KPN_I_300_R	GGACATGAACAGACACTTCTCAA			300	1.90
	Kpn_I_149	KPN_I_149_F	TTGTGAATAGTGCCGCAATAAACA	KPN_I_60_R	GAAGTCAGTGTGGCAATTCCTCAG			149	1.87
	Kpn_I_168	KPN_I_168_F	TGGTTCCAAGTCTTTGCTATTGTG	KPN_I_60_R	GAAGTCAGTGTGGCAATTCCTCAG			168	1.89
	Kpn_I_291	KPN_I_291F	TCCAATTTCATCCATGTCCCTACA	KPN_I_60_R	GAAGTCAGTGTGGCAATTCCTCAG			291	1.81
	Kpn_I_421	KPN_I_421_F	ACAACAGTCCCCAGAGTGTGATA	KPN_I_60_R	GAAGTCAGTGTGGCAATTCCTCAG			421	1.88
	APP_67	APP_67_F	TCAGGTTGACGCCGCTGT	APP_67_R	TTCGTAGCCGTTCTGCTGC			67	1.97
	APP_180	APP_67_F	TCAGGTTGACGCCGCTGT	APP_180_R	TCTATAAATGGACACCGATGGGTAGT			180	1.95
**ccfmtDNA**	***mtDNA_100***	mt_100_F	AATCCATTGTCGCATCCA	mt_100_R	GTGGCTCAGTGTCAGTTC			100	1.88
	mtDNA_417	mt_100_F	AATCCATTGTCGCATCCA	mt_417_R	GGTTGTATAGGATTGCTTGAAT			417	1.83
**ccfDNA methylation**	***Line1_153***	Line1_153_F	GAAAGGGAATTTTTTGATTTTTTG	Line1_153_R	Bio-TTTCTACATTTCCATCTAAAATACC	Line1_153_Pyro[1]	TTTTAGGTGAGGTAATGTTT	153	
	Line1_146	Line1_146_F	TTTTGAGTTAGGTGTGGGATATA	Line1_146_R	Biotin-AAAATCAAAAAATTCCCTTTC	Line1_146_Pyro[2]	GGGTGGGAGTGAT	146	
**ccfmRNA**	***GAPDH***	GAPDH_102_F	AGCGAGATCCCTCCAAAATCAAGT	GAPDH_50_R	GCAAATGAGCCCCAGCCTTCT	GAPDH_P	FAM-TGGAGTCCACTGGCGTCTTCACCACCA-BHQ1	102	1.93
	B2M	B2M_102_F	GATGTCTCGCTCCGTGGC	B2M_51_R	GGATGACGTGAGTAAACCTGAATCT	B2M_P	FAM-TGGCCTGGAGGCTATCCAGCGTACTCC-BHQ1	102	1.93
	GSTA1/GSTA2	GSTA1A2_72_F	CAAGTGCCAATGGTTGAGAT	GSTA1A2_72_R	GCTGGCAATGTAGTTGAGAA	GSTA1/A2_P	FAM-TGGTCTGCACCAGCTTCATCCCATC-BHQ	72	1.97
**ccfrRNA**	***18S_59***	18s_59_F	GACTCAACACGGGAAACCT	18s_59_R	GCTATCAATCTGTCAATCCTGTC			59	1.88
	18S_115	18s_59_F	GACTCAACACGGGAAACCT	18s_115_R	AATCGCTCCACCAACTAAGAA			115	1.82
	18S_270	18s_59_F	GACTCAACACGGGAAACCT	18s_270_R	CGGACATCTAAGGGCATCA			270	1.86
	18S_495	18s_59_F	GACTCAACACGGGAAACCT	18s_495_R	CCTCACTAAACCATCCAATCG			495	1.72
**ccfmiRNA**	***miR-93-5p***	**Qiagen Genglobe**	Genglobe ID: YP00204715						1.97
	miR-22-3p		Genglobe ID: YP00204606						1.98
	miR-320d		Genglobe ID: YP00205667						1.89
	UniSp6		Genglobe ID: YP00203954						1.94
	miR-126-3p		Genglobe ID: YP00204227						1.96
	miR-21-5-p		Genglobe ID: YP00204230						2.02
	miR-483-5p		Genglobe ID: YP00205693						1.87
	miR-122-5p		Genglobe ID: YP00205664						1.97
	miR-23a-3p		Genglobe ID: YP00204772						2.12
	miR-451a		Genglobe ID: YP02119305						1.99

[1] sequence to analyze: YGTTTTGTTTYGGTTYGYGTAYGGTGYGYGTATATAGTGGTTTGYGTTTATTGTTTGGTATTTTTTAGTGAGATGAATTYGGTATTTTAG

[2] sequence to analyze: TYGATTTTTTAGGTGYGTTYGTTATTTTTTTTTTTGATTYGGAAAGGGAATTTTTTGATTTTTTGYGTTTTTTAGGTGAGGTAA

Table S2. List of differentially expressed ccfmiRNAs in older individuals (50-68 years old) compared to younger (29-39 years old) individuals obtained from miRNA-sequencing experiments (n = 24 samples).

Name[1,2,3]	Log_2 fold change	*p*-value[4]
hsa-miR-30b-5p	2.42537514	**0.0001657**
hsa-miR-20a-3p	-2.253542281	**0.0006489**
hsa-miR-885-5p	2.741055747	**0.0006618**
hsa-miR-23b-3p	1.892802365	**0.0008746**
hsa-miR-3659	8.324176775	**0.0008763**
hsa-miR-331-3p	3.221127036	**0.001667**
hsa-let-7f-1-3p	3.140391602	**0.001709**
hsa-miR-145-5p	1.994980938	**0.002599**
hsa-miR-30c-5p	1.937215789	**0.003142**
hsa-miR-20a-5p	-1.513828246	**0.003331**
hsa-miR-98-3p	5.601527398	**0.003878**
hsa-miR-223-3p	1.628177259	**0.004753**
hsa-miR-483-5p	1.870188445	**0.00993**
hsa-miR-17-3p	-1.551445399	**0.010655167**
hsa-miR-485-3p	8.512758507	**0.011576347**
hsa-miR-194-5p	1.743633037	**0.01411762**
hsa-let-7a-3p	1.94506665	**0.014157771**
hsa-miR-96-5p	2.406025319	**0.019533804**
hsa-miR-1299	-3.205653442	**0.020078691**
hsa-miR-339-5p	1.857633497	**0.020182503**
hsa-miR-122-5p	1.960706697	**0.022174945**
hsa-miR-6886-5p	-3.489549886	**0.022577373**
hsa-miR-6783-5p	3.619604455	**0.023847049**
hsa-miR-7976	3.951321616	**0.023960188**
hsa-miR-548k	-2.182832216	**0.024134265**
hsa-miR-122-3p	7.402918509	**0.025195001**
hsa-miR-3065-5p	1.932005175	**0.025576**
hsa-miR-483-3p	2.111152602	**0.025622**
hsa-miR-122b-5p	2.395992133	**0.026727**
hsa-miR-130b-5p	3.734231782	**0.02698**
hsa-miR-4659b-3p	-4.680019796	**0.029341**
hsa-miR-1290	1.746362644	**0.031799**
hsa-miR-6891-5p	-1.922667211	**0.032058**
hsa-miR-129-5p	-1.55628419	**0.033645**
hsa-miR-7-1-3p	1.745308723	**0.033711**
hsa-miR-3157-5p	-1.652175143	**0.036797**
hsa-miR-6842-5p	-2.029795626	**0.037086**
hsa-miR-200a-5p	-1.882451547	**0.038915**
hsa-miR-874-3p	2.114546224	**0.038946**
hsa-miR-23b-5p	6.188579942	**0.040664**
hsa-miR-193b-5p	1.777379601	**0.041546**
hsa-miR-574-3p	1.646716652	**0.043435**
hsa-miR-99a-3p	3.484618912	**0.046241**
hsa-miR-136-3p	-2.058418128	**0.047277**
hsa-miR-3614-5p	-1.906795062	**0.047601**
hsa-miR-590-5p	2.040227209	**0.048984**
hsa-miR-153-3p	-5.801897743	**0.049922**
hsa-miR-93-5p	-1.321757579	**0.025363**
hsa-miR-21-5p	1.124679475	0.413288
hsa-miR-126-3p	-1.108730461	0.474223
hsa-miR-320d	1.111123834	0.51861
hsa-miR-22-3p	-1.001039013	0.994632

[1] ccfmiRNAs with a *p*-value ≤ 0.05 and fold change ≤ 1/1.5 or ≥ 1.5 are listed.
[2] Housekeeping-miRNAs (*miR-93-5p*, *miR-22-3p*, *miR-320d*), age-associated ccfmiRNAs (*miR-126-3p*, *miR-21-5p*, *miR-483-5p*) and liver-specific ccfmiRNA (*miR-122-5p*) previously analyzed by PCR are also reported in green.
[3] miRNAs with an FDR *p*-value ≤ 0.1 are underlined.
[4] *p*-values ≤ 0.05 are in bold.

Reference

Appierto, V., Callari, M., Cavadini, E., Morelli, D., Daidone, M. G., & Tiberio, P. (2014). A lipemia-independent NanoDrop((R))-based score to identify hemolysis in plasma and serum samples. *Bioanalysis, 6*(9), 1215-1226. doi:10.4155/bio.13.344
